# Silymarin and Inflammation: Food for Thoughts

**DOI:** 10.3390/antiox13010098

**Published:** 2024-01-14

**Authors:** Peter F. Surai, Anton Surai, Katie Earle-Payne

**Affiliations:** 1 Vitagene and Health Research Centre, Bristol BS4 2RS, UK; 2 Faculty of Veterinary Medicine, Trakia University, 6000 Stara Zagora, Bulgaria; 3 Faculty of Agricultural and Environmental Sciences, Szent Istvan University, H-2103 Gödöllo, Hungary; 4 Biochemistry and Physiology Department, Saint-Petersburg State University of Veterinary Medicine, 196084 St. Petersburg, Russia; 5 Faculty of Veterinary Medicine, Sumy National Agrarian University, 40021 Sumy, Ukraine; 6 Faculty of Technology of Grain and Grain Business, Odessa National Technological University, 65039 Odessa, Ukraine; 7 Healthy Food Services, Bristol BS4 2NN, UK; asurai@feedfood.co.uk; 8 NHS Greater Glasgow and Clyde, Renfrewshire Health and Social Care Centre, 10 Ferry Road, Renfrew PA4 8RU, UK

**Keywords:** silymarin, inflammation, antioxidant, oxidative stress

## Abstract

Inflammation is a vital defense mechanism, creating hostile conditions for pathogens, preventing the spread of tissue infection and repairing damaged tissues in humans and animals. However, when inflammation resolution is delayed or compromised as a result of its misregulation, the process proceeds from the acute phase to chronic inflammation, leading to the development of various chronic illnesses. It is proven that redox balance disturbances and oxidative stress are among major factors inducing NF-κB and leading to over-inflammation. Therefore, the anti-inflammatory properties of various natural antioxidants have been widely tested in various in vitro and in vivo systems. Accumulating evidence indicates that silymarin (SM) and its main constituent silibinin/silybin (SB) have great potential as an anti-inflammation agent. The main anti-inflammatory mechanism of SM/SB action is attributed to the inhibition of TLR4/NF-κB-mediated signaling pathways and the downregulated expression of pro-inflammatory mediators, including TNF-α, IL-1β, IL-6, IL-12, IL-23, CCL4, CXCL10, etc. Of note, in the same model systems, SM/SB was able to upregulate anti-inflammatory cytokines (IL-4, IL-10, IL-13, TGF-β, etc.) and lipid mediators involved in the resolution of inflammation. The inflammatory properties of SM/SB were clearly demonstrated in model systems based on immune (macrophages and monocytes) and non-immune (epithelial, skin, bone, connective tissue and cancer) cells. At the same time, the anti-inflammatory action of SM/SB was confirmed in a number of in vivo models, including toxicity models, nonalcoholic fatty liver disease, ischemia/reperfusion models, stress-induced injuries, ageing and exercising models, wound healing and many other relevant model systems. It seems likely that the anti-inflammatory activities of SM/SB are key elements on the health-promoting properties of these phytochemicals.

## 1. Introduction

Inflammation is an important defense mechanism in humans and animals responsible for creating hostile conditions for pathogens and preventing the spread of tissue infection. The main task of acute inflammation is related to the elimination of infectious agents, repair of damaged tissues with the following termination of the inflammation and the re-establishment the homeostasis (resolution; [1]). However, when inflammation resolution is delayed or compromised due to misregulation of the immune response, and the process proceeds from the acute phase to chronic inflammation, this can lead to the development of various chronic illnesses, including atherosclerosis, cancer, obesity, asthma, chronic obstructive pulmonary disease, multiple sclerosis, inflammatory bowel disease and neurodegenerative disease (Alzheimer’s disease, Parkinson’s disease, amyotrophic lateral sclerosis), and autoimmune disorders, such as rheumatoid arthritis, systemic lupus erythematosus, psoriasis and celiac disease [1,2]. There are a range of stress factors in human life and animal/poultry production (Table 1), which can cause excessive inflammation, compromising health and promoting various diseases.

It seems likely that disturbances in redox homeostasis due to the misregulation of the antioxidant defence network are important elements in over-inflammation in humans and farm animals/poultry. For the last decade, antioxidant and anti-inflammatory properties of SM/SB have been extensively studied [2,5,6,7]. Therefore, the main aim of this paper is to present an updated view on the anti-inflammatory properties of silymarin/silybin with specific emphasis on the molecular mechanisms of protective actions of the phytochemical.

## 2. Molecular Mechanisms of Inflammation

**Figure 1:**
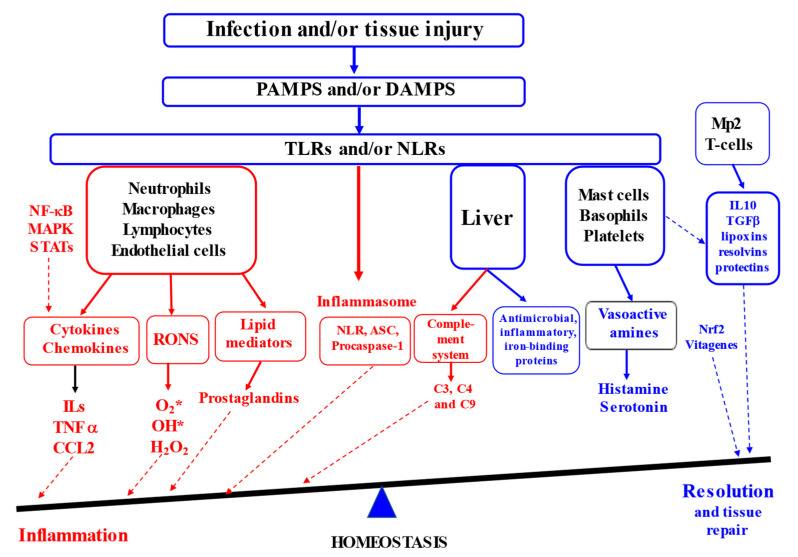
Inflammation and resolution (Adapted from [2,3,13,14,15]). (ASC—the adaptor molecule apoptosis-associated speck-like protein containing a CARD; CCL2—chemokine (C–C motif) ligand 2; DAMPS—damage-associated molecular patterns; IL-10—interleukin 10; MAPK—mitogen-activated protein kinases; STAT—signal transducer and activator of transcription; Mp2—type 2 macrophages; NLR—Nucleotide oligomerization domain (NOD)-like receptors; PAMPS—pathogen-associated molecular patterns; TGF—transforming growth factor; RONS—reactive oxygen and nitrogen species; NLR—nucleotide-binding domain, leucine-rich repeat containing; TNFα—tumour necrosis factor alpha TLRs—Toll-like receptors). Red colour indicates factors contributing to inflammation and blue colour covers factors contributing to resolution.

Inflammation is known to be a complex biological response that the body uses to defend itself against harmful stimuli, including pathogens, damaged cells or irritants [2,8]. At the tissue structural level, inflammation is usually associated with redness, swelling, heat, pain and loss of tissue function [9]. Furthermore, increased vascular permeability, enhanced leukocyte recruitment and accumulation associated with inflammatory mediator release are among the major microcirculatory events related to the inflammatory responses [6,10,11]. It is generally accepted that inflammatory responses are initiated in response to the host recognition of inflammatory signals in the form of either pathogen-associated molecular patterns or damage-associated molecular patterns by various pattern recognition receptors (PRR [12]) in the innate immune system. This includes surface Toll-like receptors (TLRs) and cytoplasmic NOD (nucleotide-binding oligomerisation-domain)-like receptors (NLRs), leading to the production of inflammatory cytokines, chemokines and prostaglandins. Activated immune cells (e.g., neutrophils, macrophages, lymphocytes and mast cells) are responsible for the synthesis and release of a large number of cytokines and chemokines (TNF-α, IL-6, IFN-γ, NO, MCP-1, CXCL10, etc. [3]). It is known that neutrophils are leaked from blood vessels as a result of vasodilatation due to the secretion of vasoactive amines (histamine and serotonin) by mast cells, basophils and platelets being the first immune cells to appear at the place of infection or injury. Further, the neutrophils induce re-localisation and activation of macrophages to arrive at the site of infection or injury. Therefore, the synthesis and secretion of communicating molecules, including cytokines, by activated phagocytic cells (e.g., neutrophils and macrophages) control the process of inflammation associated with the activation of various transcription factors and enzymes (e.g., NF-κB, STAT, AP-1, HIF-1-α, iNOS, COX-2 and NLRP3 inflammasome). When the major task of the pathogen destruction and/or injury repair is completed and cellular debris is removed, the resolution of inflammation takes place, e.g., the acute inflammatory response is terminated (Figure 1, [3]).

Therefore, acute inflammation is an essential part of the immune system’s defence strategy, contributing to the healing process. However, when inflammation resolution is dysregulated, chronic inflammation in the heart, pancreas, liver, kidney, lung, brain, intestinal tract and reproductive system leads to tissue damage and the development of various diseases in humans and animals [9,16].

## 3. Oxidative Stress and Inflammation

It is generally accepted that oxygen is a vital element for eukaryote energy production in mitochondria; however, this process is not 100% efficient and some electrons can escape from the electron transport chain (ETC), forming free radicals. In addition, phagocyte cells produce free radicals and use them as a weapon to kill pathogens. In general, major free radicals (O_2_*, OH*) and some non-radical molecules (H_2_O_2_) are combined under reactive oxygen species (ROS). For four decades, ROS-related research has generated important information, essential for understanding not only the detrimental but also beneficial role of free radicals in cell signalling, stress adaptation as well as in other physiological processes. Of note, the benefit or harm of ROS generally depends on the level of their production and efficiency of their detoxification by the antioxidant defence network. In fact, disturbances or redox balance and oxidative stress are responsible for the development of a range human diseases [17]. The antioxidant defence network in various tissues is based on internally synthesised (superoxide dismutase, catalase, glutathione peroxidase, thioredoxin reductase and other selenoproteins, glutathione, thioredoxin, ascorbic acid, ubiquinone, taurine, carnitine, etc.) and externally supplied antioxidants (vitamin E, carotenoids, polyphenols, taurine, carnitine, etc.). All the antioxidants in the body work together, providing an effective antioxidant defence and maintaining the redox balance. The antioxidant defences are regulated via various transcription factors, including Nrf2 and NF-κB, and the vitagene network [4].

The concept of oxidative stress as an imbalance between oxidants and antioxidants and oxidative stress responses was developed by Sies in 1985 [18] and, recently, it has been updated [4,17] to include current development and understanding of the topic. This can be briefly summarised as follows. Animals and humans manage stress via adopting various mechanisms, generally called “stress response” (SR), associated with the induction of various genes encoding the synthesis of various cyto-protective molecules. Depending on the conditions, SR can be immediate, lasting from a few seconds to several hours and associated with receptor-mediated intracellular signalling, or it can be delayed with the involvement of various modulators and downstream effectors [19]. There are at least eight stress response pathways responsible for stress sensing and the development of an adequate response, including oxidative stress response (OSR), heat shock response, unfolded protein response, DNA damage response, hypoxia-induced stress response, inflammation stress response, energy stress response and nutritional stress response [4]. Interestingly, all eight stress responses are interrelated, and oxidative stress response can be placed at the centre of stress response creation. Therefore, to deal with the oxidative challenge and to maintain redox homeostasis, a stress response is initiated including the activation of gene expression in defence systems. There are two major “master regulators” of the stress response, including the Nrf2/Keap1 and the NF-κB/IκB systems [20,21,22,23]. In fact, oxidative stress response is associated with both transcription factors to be translocated to the nucleus and create protective, but in many cases opposite, responses. On the one hand, Nrf2 activates a great number of genes responsible for the synthesis of an array of protective antioxidant molecules [20]. On the other hand, NF-κB induces the expression of genes associated with inflammation and immunity [21]. The stress response also includes various repair programs as well as removal/cell death programs, including autophagy, mitophagy, apoptosis, necroptosis, ferroptosis, etc. [4]. In general, redox balance maintenance and cell signalling are shown to be integrated with main homeostatic mechanisms at the molecular, organellar, cellular, tissue and organismic levels [18], and they are tightly integrated with the vitagene network and various transcription factors [4].

In general, the inflammatory process is associated with oxidative stress. On the one hand, ROS production in macrophages (so called oxidative burst) is an essential process, and they are used as a powerful weapon to kill pathogens and, therefore, activated macrophages are very efficient at recognising, phagocytosing and killing pathogens in phagolysosomes [24]. On the other hand, excess or uncontrolled ROS production can damage phagocyte cells and decrease their protective efficiency. However, ROS also play signalling roles in macrophage polarisation and inflammation resolution. Therefore, if the inflammation is not properly controlled and not terminated in timely way, it can become a chronic state, detrimentally affecting tissue integrity and wound healing. This is associated with the induction of the transcription factor NF-κB, which regulates the expression and synthesis of inflammatory mediators [12]. Once the infection is eliminated, NF-κB activity should drop to its basal level; however, in pathological inflammatory processes, an incorrect termination of NF-κB signalling is observed [25]. It has been shown that silymarin possesses antioxidant and anti-inflammatory properties, being an important phytochemical used to regulate inflammatory responses [2,5,6].

## 4. Chemical Composition and Structure of Silymarin

**Figure 2:**
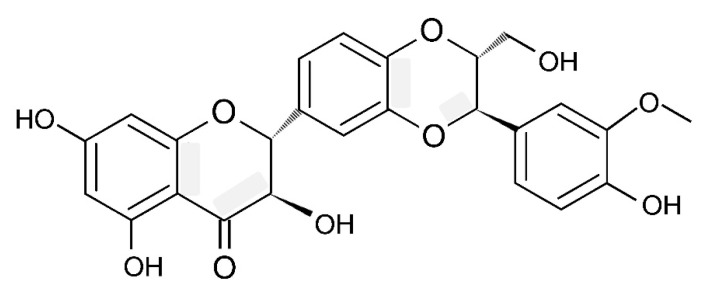
Silybin structure.

Silymarin (SM) is a standardised extract from the milk thistle *Silybum marianum* containing a complex of flavonolignans (silybin A, silybin B, isosilybin A, isosilybin B, silychristin, isosilychristin, silydianin, and the flavonoids taxifolin, quercetin and kaempferol) as its main constituents (65–80% [26,27,28]). To obtain flavonolignans, the seed meal is usually subjected to successive washes with petroleum ether to remove lipids, and flavonolignans are then extracted with ethanol [29]. SM is sown to contain silybin A and B as major active constituents and also includes isosilybin A and B (about 5%), silychristin A (7–10%), silydianin (10%) with a smaller amount of taxifolin (2–5%) and 2,3-dehydroflavonolignans (≤3%; [27]). It is important to underline that the chemical composition of SM is quite variable and depends on the variety of *S. marianum* [30], plant growing conditions such as soil composition, climatic conditions as well as the techniques for plant processing [27,31,32,33]. Silybin has a molecular weight of 482.441, a molecular formula of C_25_H_22_O_10_ and a CAS Number of 22888-70-6 (PubChem, [34], Figure 2).

Importantly, in scientific literature terms, silybin and silibinin are used as synonyms [35]. As can be seen from Figure 2, in the molecular structure of silybin, there are two domains, connected by a 1,4-dioxane ring. The first one comprises the flavononol group, and the second domain is related to the phenylpropanoid group. It is reported that silybin is characterised by five hydroxyl groups, which are the main targets for derivatisation [34]. Natural silybin is found to be a mixture of silybin A and B, with a ratio of these two stereoisomers of about 3:7 [27]. Interestingly, in older studies, this ratio was shown to be 1:1 [36]. In fact, this ratio depends on the source and processing conditions of SM [35]. Therefore, a high degree of compositional variability in SM [35] is believed to be responsible for experimental results’ variability.

## 5. Silymarin and Inflammation: In Vitro Studies

The anti-inflammatory properties of silymarin (SM) or its main constituent, namely silibinin/silybin (SB), have been proven in various in vitro systems based on cultivated cells including immune cells and other types of cells. Macrophages and monocytes are believed to be important players in the innate immune response and vital regulators of inflammation. They are effective drivers of innate immunity, performing phagocytosis and initiating inflammation, inducing the adaptive immune response, leading to the resolution of inflammation and homeostasis re-establishment [24].

### 5.1. Macrophages

It is well established that macrophages (MPs) play a critical role in tissue development/homeostasis, being responsible for the tissue surveillance to detect infection or injury as well as monitoring tissue changes [24]. There is a growing consensus that the recognition of various stimuli by MPs is executed by a great variety of receptors, including cytokine receptors, PRRs and phagocytosis receptors [37]. There is also mounting evidence that depending on conditions, macrophages can be characterised by various activated phenotypes (e.g., the M1 and M2 phenotypes). In fact, M1 macrophages are characterised by pro-inflammatory activities due to secreting important cytokines, chemokines, including TNF-α, IL-1β, IL-6, IL-12, IL-23, CCL4 and CXCL10, and ROS, including O_2_*, OH* and H_2_O_2_ [8,38]. This leads to inflammation, phagocytosis and oxidative burst with pathogen destruction/killing. In marked contrast, M2 macrophages produce anti-inflammatory cytokines (e.g. IL-4, IL-10, IL-13, TGF-β, etc.) and lipid mediators (lipoxins, resolvins, protectins, etc.), leading to the resolution of inflammation [24,39]. Within this scenario, to deal with infection, at the early stage, there is a macrophage phenotype switch from a homeostatic function to proinflammatory phenotype. A growing body of evidence indicates that once the pathogen is dealt with, a switch to an anti-inflammatory/pro-resolving macrophage phenotype takes place in order to limit tissue damage, terminate the inflammatory response/resolve inflammation and re-establish homeostasis [24].

Growing evidence also indicates that the M1/M2 classification of MPs seems to be out of date, since it does not take into account the complexity of the physiological or the pathophysiological contexts and the diversity of the stimuli activating MPs [37]. However, to simplify this issue, we keep the main principles of polarisation as they were previously characterised. Of note, the misregulation of this macrophage polarisation could lead to over-inflammation associated with the development of various inflammation-related states/diseases.

Several studies based on LPS-stimulated macrophages clearly demonstrated the anti-inflammatory properties of SM/SB. For example, SM (50 μg/mL) was reported to supress morphological changes in LPS-stimulated RAW264.7 mouse macrophages due to the inhibition of the NF-κB pathway, including a reduction in the nuclear translocation as well as decreasing the transactivation activities of NF-κB [40]. Similarly, in cultured macrophages, SM (~10–40 μM) was observed to supress LPS-induced NO generation and IL-1β expression [41]. In a similar model system, SB (40 μM) was indicated to inhibit inflammatory indexes (NF-κB, IL-6 and TNF-α). Importantly, the combination of SB and thymol (40 μM and 120 μM, respectively) demonstrated a more pronounced protective effect in comparison to the single usage of SB [42]. On the same line, the increased anti-inflammatory actions of SB and capsaicin combination in LPS-induced RAW264.7 cells was due to the suppression of NF-κB and MAPK activation [43]. The in vitro results obtained with RAW264.7 cells showed that SB suppressed RANKL-induced nuclear factor of activated T cells 1 (NFATc1) induction and translocation via the NF-κB and MAPK signalling pathways. Furthermore, SB decreased the inflammatory cytokine level and oxidative stress in LPS-stimulated human gingival fibroblasts [44]. Silymarin-functionalized selenium nanoparticles were reported to be more effective in comparison to silymarin in preventing LPS-induced inflammatory response (NF-κB, IL-1β and TNF-α) in RAW264.7 cells through downregulation of the PI3K/Akt/NF-κB pathway [45].

In a model of toxicant-induced macrophage injury, the pretreatment of mouse macrophages with SM (100 μM) was observed to decrease paraquat (PQ)-induced cytotoxicity, simultaneously with an enhancement in the expression of Trx and antioxidant enzymes (SOD and GPx), the inhibition of oxidative stress and the prevention of the TXNIP and NLRP3 inflammasome activation, simultaneously with a reduction in proinflammatory cytokine (IL-1β and IL-18) secretion [46]. Indeed, in mice, SB was effective in preventing the formation of the NLRP3 inflammasome complex via the NAD^+^/SIRT2 pathway [47]. In these settings, other stimulating antigens/agents were also successfully employed. For example, in *H. pylori*-stimulated macrophages, SB (103.6 μM) was able to suppress the production of pro-inflammatory mediators, including TNF-α, IL-10 and IL-6, by 100%, 70.3% and 33.9%, respectively [48].

There is considerable evidence that SM treatment of primary mouse macrophages induced M2 polarisation with decreased levels of pro-inflammatory cytokine (TNF-α and IL-1β) production and enhanced the levels of anti-inflammatory (IL-10) cytokines [49]. In this regard, it has been demonstrated that SB suppressed inflammation by promoting M2-type macrophage polarisation associated with the secretion of anti-inflammatory factors (CD206, IL-10) and the suppression of the secretion of inflammatory factors (IL-1β, iNOS; [50]).

### 5.2. Mononuclear Cells

Accumulated knowledge indicates that monocytes comprise about 5–10% of all blood immune cells with a life span of about 1–3 days, and during an inflammatory process, they can differentiate either into inflammatory macrophages or dendritic cells [24]. There is evidence suggesting that in CD14+/− human monocytes challenged by *Paracoccidioides brasiliensis* (Pb18), SB showed anti-inflammatory and anti-fibrotic effects due to the suppression of p65 NF-κB activation [51]. In a subsequent study, treatment with SB (50 μM) mononuclear cells (lymphocytes and monocytes) obtained from preeclamptic women was demonstrated to decrease LPS-stimulated NF-κB activation and to inhibit the production of such inflammatory cytokines as TNF-α and IL-1β [52]. It has been recognised that in HMC-1 human mast cells, SB (50–200 μM) can suppress pro-inflammatory cytokine (TNF-α, IL-6, and IL-8) production by inhibiting the NF-κB signalling pathway [53]. These observations are consistent with an observation that SB reduced histamine release from rat peritoneal mast cells (RPMCs) induced by compound 48/80 or anti-DNP IgE. It is noteworthy that SB also decreased the secretion of pro-inflammatory cytokines, including TNF-α and IL-6 in RPMC [54].

It is of considerable interest that SM treatment (0.1 mM) of LPS-stimulated peripheral blood mononuclear cells from healthy donors was associated with a reduction in the production of such important proinflammatory cytokines as IL-1α, IL-1β, IL-6, TNF-α and IFN-γ [55]. It is of interest that SM also has inhibitory actions on chemokine production, including CCL2, CCL5 and MIP-1, which could affect the recruitment of monocytes and neutrophils to sites of local inflammation and regulate inflammation [55]. Most importantly, it confirmed that the anti-inflammatory action of SM was associated with a decrease in NF-κB activation.

In various cell cultures in vitro, the anti-inflammatory activities of SM were also demonstrated. Indeed, SM treatment was associated with the inhibition of multiple pro-inflammatory mRNAs and signalling pathways, including NF-κB and FOXO [56]. This SM action was shown to be partially AMPK dependent. Importantly, in primary human immune cells, SM (80 μM) also demonstrated broad anti-inflammatory and immunoregulatory activity, leading to the inhibition of pro-inflammatory cytokine expression and production and modulation of T cells [57]. Results from the same study revealed that SM treatment of T cells obtained from chronically infected, HIV-positive patients suppressed the expression of T-cell activation and exhaustion markers on CD4+ and CD8+ cells. These results have been interpreted as indicating that SM can modulate multiple metabolic pathways and cause the suppression of inflammation in diverse immune cell types.

In line with that discussed above, SB treatment (10 and 50 μM) of equine peripheral blood mononuclear cells produced anti-inflammatory effects associated with the mitigation of LPS-induced production of proinflammatory cytokines, including TNF-α, IL-1β, IL-6 and IL-8 [58]. This view is supported by several observations, including a study conducted by Lovelace et al. [56] using transcriptional profiling, metabolomics and signalling studies in T-cell lines and the human liver. Overall, SM demonstrated anti-inflammatory properties as a result of the suppression of expression of various pro-inflammatory cytokines and signalling pathways, including NF-κB and FOXO. This was also illustrated in monocytes from preeclamptic women treated with SB (50 μM) and leading to downregulation of the endogenous activation of NF-κB and to the inhibition of the synthesis of pro-inflammatory cytokines (IL-1β, IL-6, IL-8, IL-12p70, IL-23 and TNF-α), in comparison to control cultures not treated with SB. In the meantime, in monocytes from preeclamptic women, SB also induced in vitro M2-like phenotype polarisation [59]. It seems highly likely that there are other mechanisms of anti-inflammatory actions of SM/SB on lymphocytes. For instance, T lymphocytes from female and male healthy donors treated with SB demonstrated a reduction in expression of pro-inflammatory cytokines (IL-17 and TNF-α), through ERβ binding and the upregulation of ERβ expression [60].

### 5.3. Epithelial Cells

In agreement with the above reports, SM (10–20 μM) was found to attenuate cigarette smoke-induced upregulation of inflammatory cytokines (TNF-α, IL-6 and IL-8) in human bronchial epithelial cells and decreased levels of TNF-α in cigarette smoke-exposed mice [61]. SM was also demonstrated to have inhibitory effects on obesity-induced growth and ROS production associated with decreased production of proinflammatory IL-6 and IL-1β in HepG2 cells exposed to sera from obese individuals [62]. In NCI-H292 airway epithelial cells, stimulated by silica dioxide nanoparticles, SB was reported to mitigate elevated inflammatory indexes (TNF-α, IL-6 and IL-1β) associated with a reduction in TXNIP, MAPKs and AP-1 expression [63]. Of interest, the pretreatment of human umbilical vein endothelial cells (HUVECs) with SB was observed to reduce the TNF-α-induced gene expression of such proinflammatory genes as IL-6 and MCP-1 [64]. Silibinin was demonstrated to mitigate LPS-induced inflammation (TNF-α) in porcine mammary epithelial cells via the regulation of the mTOR/NF-κB signalling pathway, including the attenuation of increased expression of (phosphorylated) p-NF-κB p65, p-IκB-α and p-MAPK p38 [65].

### 5.4. Skin and Bone Cells

Growing evidence indicates that SM/SB can decrease inflammatory responses in the skin cells. For instance, skin injury (skin cells and SKH-1 hairless mouse skin) induced by sulfur mustard analog was reported to be ameliorated by SB (10 μM in vitro and 1 mg with topical application) as a result of affecting various pathways associated with oxidative stress and inflammation, including NF-κB and AP-1 [66]. More recently, an active component of SM (2,3-dehydrosilybin, 10 and 15 μM) was demonstrated to protect LPS-challenged human dermal fibroblasts by dose-dependently diminishing IL-6 and IL-8 secretion into the surroundings, with simultaneous upregulation of IL-8 mRNA associated with NF-κB and AP-1 activation [25]. According to a recent study, LPS treatment of keratinocytes can induce the inflammatory response and release of pro-inflammatory cytokines (IL-1α and IL-6) and the chemokine IL-8. In this context, SB treatment (15 μM) of human epidermal keratinocytes was associated with the downregulation of the LPS-induced production of proinflammatory cytokines, including IL-1α, IL-6 and IL-8 [67]. In human chondrocytes in vitro, SB was demonstrated to inhibit the expression of IL-1β-induced inflammatory markers, including COX-2, iNOS, NF-κB, NO, PGE2, TNF-α and IL-6 [68]. Similarly, SB (0.42 mM) regulated intracellular signalling to reprogram the inflammatory response in osteoblast SB [69]. In summary, SB was shown to be protective against osteoarthritis (OA) through inhibiting the inflammatory response and cartilage matrix degradation in vitro (in human OA chondrocytes) and in vivo (in mice OA models).

### 5.5. Connective Tissue Cells

By inhibiting the NF-κB pathway, SB (50–150 mg/kg) was able to supress the production of inflammatory cytokines (TNF-α, IL-1β and IL-6) in rheumatoid arthritis fibroblast-like synoviocytes [70]. Improved AO defences decreased oxidative stress and ameliorated H_2_O_2_-induced injury in fibroblast cells due to SM treatment [71]. Additional evidence exists that the protective effects of SM are likely to be condition-dependent. For instance, treatment of LPS-challenged human dermal fibroblasts with an SM component, namely 2,3-dehydrosilybin (DHS), could have both beneficial and detrimental effects. Indeed, DHS was indicated to decrease IL-6 and IL-8 secretion. However, it could also significantly upregulate IL-8 mRNA associated with NF-κB and AP-1 activation [25]. Of note, DHS was demonstrated in various in vitro assays to significantly diminish NO production and TNF-α and IL-6 release in a dose-dependent manner [72].

### 5.6. Cancer Cells

Recent studies have focused on the anti-inflammatory action of SM/SB in cancer cells. For instance, human colorectal cancer cells (SW480, LoVo, and HT29) treated with SB (50–200 µM) demonstrated reduced TNF-α-induced NF-κB activation together with decreased nuclear levels of both p65 and p50 sub-units. This was associated with an increased IκBα level and decreased phospho-IκBα [73]. In a similar fashion, SM-treated THP-1 cells were characterised by the inhibition of caspase-1 cleavage and IL-1β and reduced TNF-α production [74]. The inclusion of SB into the incubation medium was also reported to show anti-inflammatory action in monosodium urate-activated THP-1 cells. The protective activity of SB was evidenced by the diminished inflammation-related gene expression of NLRP1/NLRP3 inflammasomes and TLR4/NF-κB pathway as well as through the reduced production of pro-inflammatory cytokines, including IL-1β, IL-18 and TNF-α [75]. The anti-inflammatory actions of SB, as evident from the suppression of NF-κB and STAT3 with decreased expression of COX-2 and iNOS, were clearly demonstrated in human gastric cancer MKN-1 cells and in the stomach of C57BL/6 mice infected with *H. pylori* [76]. Interestingly, SB was shown to have a cytoprotective effect in IPEC-1 cells, including mitochondrial integrity, and increased the expression of anti-inflammatory TGF-β. However, in great contrast, in tumour intestinal CaCo-2 cells, SB was able to increase apoptosis and significantly reduced the expression of pro-inflammatory cytokines [77]. Furthermore, in a *Drosophila melanogaster* model, SM was demonstrated to relieve intestinal inflammation caused by dextran sulfate sodium via modulating the c-Jun N-terminal kinase (JNK) signalling pathway [78]. In a rat model of experimentally induced renal carcinogenesis, treatments with SM (150 mg/kg b. w.) or SB (5 mg/kg b. w.) for 9 weeks were shown to suppress proinflammatory mediators (NF-κB, p65, I*κ*B*α* and IL-6) and inhibited the PI3K/Akt pathway. Furthermore, the phytochemicals induced the expressions of PPARs, Nrf2 and IL-4 simultaneously with the downregulation of apoptotic proteins p53 and caspase-3 and upregulation of antiapoptotic mediator Bcl-2 [79].

### 5.7. Other Cell Types

Recently, SB (50 and 100 μM) has been reported to show a significant protective anti-inflammatory effect (decreased expression of IL-1β, IL-6 and TNF-α and increased expression of IL-10) in the retinal ganglion cells damaged by blue-light-emitting diodes due to the activation of the MEK/ERK/CREB pathway [80]. In a model of myocardial I/R injury, the SB-associated cardioprotective effect was illustrated to be a result of decreased oxidative stress, apoptosis and inflammatory response due to downregulation of the NF-κB pathway. In fact, SB was effective in inhibiting NF-κB signalling (inhibiting IκBα degradation, IKKα phosphorylation and p65 NF-κB nuclear translocation) during hypoxia/reperfusion in embryonic rat cardiomyocytes [81]. The human periodontal ligament cells (PDLs) were treated with SB (10, 20 and 40 μM) in the presence of LPS. The results clearly demonstrated that SM treatment was associated with reduced levels of pro-inflammatory mediators, including NO, PGE_2_, IL-6, TNF-α, MMP-1 and MMP-3, and improved AO defences by enhancing the activities of SOD and increasing GSH levels [82]. The anti-inflammatory protective effects of SB were evaluated in a rat ligature-induced periodontitis model and an LPS-stimulated human periodontal ligament cell model. In such stress conditions, SB was found to maintain the expression of Nrf2 and attenuated lipid, protein and DNA oxidative damage in the periodontal lesion area. In both in vitro and in vivo models, SB demonstrated strong anti-inflammatory properties, indicative of the suppression of expression of NF-κB and NLRP3 associated with a downregulation in the levels of proinflammatory cytokines, including IL-1β, IL-6 and TNF-α [83].

## 6. Silymarin and Inflammation: In Vivo Studies

Various disease models were used to demonstrate the attenuation of inflammation by SM/SB treatment. On one hand, extensive research efforts during the last two decades clearly demonstrated that the protective effects of SM/SB were associated mainly with inhibition of the NF-κB pathway and downregulation of pro-inflammatory cytokines, including TNF-ɑ and IL-1β [53,84]. On the other hand, SM/SB can induce transcriptional factors (e.g., Nrf2), regulating cellular defence against inflammatory and oxidative challenges [85,86]. It should be noted that SB was studied in a mouse model of concanavalin A (ConA)-induced, T-cell-dependent hepatitis and was demonstrated to possess immunomodulating properties in vivo, suppressing the intrahepatic expression of proinflammatory mediators, such as TNF-α, INF-γ, IL-4, IL-2, iNOS and NF-κB, with the simultaneous augmentation of synthesis of anti-inflammatory IL-10 [87].

### 6.1. Nonalcoholic Fatty Liver Disease (NAFLD)/Steatohepatitis/Diet-Induced Obesity

A large body of evidence indicates that NAFLD is associated with metabolic syndrome and type 2 diabetes, having important links to inflammation. The disease is known to include a range of pathological conditions, ranging from relatively benign steatosis characterised by ectopic lipid storage in the liver, to nonalcoholic steatohepatitis (NASH) associated with inflammation as a major factor in tissue remodelling and functional impairment with possible progression to hepatocellular carcinoma [88]. In this sense, parenchymal cell injury and death are considered to trigger inflammation and tissue fibrosis [89].

Several studies demonstrated the anti-inflammatory protective actions of SM/SN in various models of NAFLD. Firstly, in a mouse model of experimental nonalcoholic steatohepatitis, SB (20 mg/kg intraperitoneally for 4 weeks) was reported to affect lipid homeostasis and to supress NF-κB activation in the liver [90]. Secondly, in high-fat diet (HFD)-induced nonalcoholic steatohepatitis in vivo and in fat-laden human hepatocytes in vitro, SM was illustrated to suppress hepatic inflammation, oxidative stress and apoptosis [91]. Thirdly, in a similar model system, SB was found to ameliorate O-linked β-N-acetylglucosamine O-GlcNAcylation associated with the NF-κB signalling pathway and led to decreased inflammation [92] and to enhanced AO defence associated with the upregulation of Nrf2, suppression of NF-κB signalling and decreased expression of proinflammatory cytokines to ameliorate hepatic steatosis and fibrosis [93]. In addition, in mice with HFD-induced nonalcoholic fatty liver disease, SB was able to suppress NLRP3 inflammasome assembly through the NAD^+^/SIRT2 pathway. This was confirmed by the disappearance of the anti-inflammatory effect of SB as a result of SIRT2 silencing or due to usage of the SIRT2 inhibitor AGK2 [23]. Importantly, SB decreased liver inflammation and inhibited NF-κB translocation into the nucleus through increased SIRT2 expression and promotion of p65 deacetylation [94].

Additional information related to the anti-inflammatory actions of SM/SM was obtained with various models of diet-induced obesity. For instance, SM (60 mg/kg) was illustrated to reduce inflammation (TNF-α, IL-1β and IL-6) and to mitigate liver damage and insulin resistance in a model of HFD-induced obesity mice [95]. In another study, SM treatment was also effective in attenuating insulin resistance, dyslipidaemia and inflammation in the liver of obese mice associated with inhibited NF-κB signalling [96]. Accumulated data confirmed the ameliorative effects of SM in adipose tissue inflammation (decreased expression of IL-1β and TNF-α and increased expression of IL-10 and adiponectin) in an HFD-induced obesity model in mice [97]. Recent studies have also indicated that milk thistle seed cold press oil was able to improve antioxidant defences (SOD1, HO-1 and SIRT1) and attenuated inflammation markers (IL-6 and NF-κB) in a mouse model of dietary-induced obesity [98]. In a recent meta-analysis of 26 randomized controlled trials involving 2375 patients, the main findings were as follows: SM significantly reduced the levels of TC, TG, LDL-C, fasting insulin (FI) and homeostatic model assessment of IR (HOMA-IR) and increased the level of HDL-C. Furthermore, SM was proven to attenuate liver injury and to decrease the levels of fatty liver index and fatty liver score. In addition, SM was able to improve hepatic steatosis [99].

### 6.2. Toxicity Models

#### 6.2.1. CCl_4_

Carbon tetrachloride (CCl_4_), an important industrial solvent and refrigerant production intermediate, is a highly toxic chemical causing oxidative stress in the liver. Accumulating evidence indicated that the metabolism of CCl_4_ via CYP2E1 with the production of ROS is an important mechanism of its toxicity. It is widely accepted that CCl_4_ can compromise the antioxidant defence network of various tissues due to the inhibition of AO enzymes (SOD, GPx and CAT), leading to oxidative stress and hepatic injuries [21]. Classically, inflammatory responses usually exacerbate chemically induced hepatotoxicity [100].

Multiple studies have reported the potential of SM/SB to control CCl_4_-induced inflammation. For instance, reduced inflammation (IL-6, MAPK, NF-κB) and liver fibrosis in CCl_4_-treated rats was observed due to SM (50 mg/kg BW for 10 weeks) treatment [101]. Furthermore, in a rat model of CCl_4_-induced liver fibrosis, SM (100 mg/kg) demonstrated anti-inflammatory (IL-6) and antioxidant (GSH and MDA) protective activities [102]. The attenuation of inflammation, oxidative DNA damage, apoptosis and fibrosis were considered molecular mechanisms of the protective effects of SM (200 mg/kg) against CCl_4_-induced hepatotoxicity [103]. This was associated with ameliorating the CCl_4_-induced increased expression of TNF-α, TGF-β1 and MCP-1, simultaneously with decreasing intrahepatic monocytes [104]. A more recent report showed that SM and SB were able to downregulate transcription factors (e.g., NF-κB) and proinflammatory cytokines (IL-6), simultaneously with the suppression of PI3K/Akt signalling and induction of Nrf2 and PPARγ expressions in a rat model of chemically induced (the diethylnitrosamine/2-acetylaminofluorene/CCl_4_) renal carcinogenesis [79]. Similarly, reduced IL-6 levels and increased levels of IL-4 as well as the inhibition of NF-κB expression in rat kidney due to SM/SB treatment were associated with protection against chemically induced renal carcinogenesis [79]. In male rats, dietary SM (100 mg/kg body weight) provided after or before and during CCl_4_-treatment was able to decrease toxicosis-induced expression of NF-κB, as well as of pro-inflammatory cytokines, including TNF-α, IL-6 and TGF-β and COX2 in the liver. This was associated with the restoration of AO defences (GSH, GPx, SOD) and improved liver functions and lipid profiles [105]. Importantly, a synergistic hepatoprotective effect of combined usage of Lachnum polysaccharide (LEP-2b) and SM was found to be associated with decreased hepatic inflammation (TNF-α, IL-6 and IL-1β) and enhanced AO defences (SOD, CAT, GPx, GSH, MDA and T-AOC) in a mouse model of CCl_4_-induced liver toxicity [106].

#### 6.2.2. Other Toxicants

SM (100 mg/kg, intraperitoneally, 2 h before toxicant treatment) was shown to mitigate maneb (MB)- and PQ-induced activation of inflammatory mediators, including iNOS, TNF-α and IL-1β, in the rat liver [107]. Furthermore, bleomycin-induced pulmonary toxicity and lipid peroxidation in mice were demonstrated to be ameliorated by SM, as evidenced by redox balance maintenance (GSH, GST and CAT) and the regulation of pro-inflammatory cytokines [108]. Similarly, 12-O-tetradecanoylphorbol-13-acetate-induced skin inflammation (IL-1β, IL-6, TNF-α, COX-2 and NF-κB) in experimental mice was dose-dependently downregulated by SB [109]. Importantly, SM (200 mg/kg for 10 days) also mitigated bisphenol A-induced hepatotoxicity in mouse, as indicated by decreased levels of pro-inflammatory cytokines (IL-6 and TNF-α) and reduced ultrastructural injuries [110]. The proinflammatory action of triptolide-induced acute hepatotoxicity in rats was dose-dependently ameliorated by SM (50, 100 and 200 mg/kg for 7 days) by inhibiting the production of pro-inflammatory cytokines (TNF-α, IL-6 and IL-1β) in the liver [111]. A reduction in neutrophil infiltration associated with decreased expression of inflammatory cytokines (TNF-α, IL-1β, IL-12) and reduced iNOS was observed in a model of APAP-induced hepatotoxicity in mice as a result of SM (100 mg/kg, once daily for three days) treatment [112]. In a similar manner, SM (50 and 200 mg/kg for 21 days) was demonstrated to dose-dependently mitigate functional damage to the liver and kidneys simultaneously with an improvement in AO defences and reduced inflammation in a model of cyclosporine A-induced hepatorenal toxicity in rats [113]. Importantly, docetaxel-induced brain and sciatic nerve injuries were also ameliorated by SM as a result of enhancements in the antioxidant defence network (GSH, SOD, CAT, GPx, HO-1 and Nrf2) and inhibition of inflammation (NF-κB and TNF-α) and apoptosis [114].

The protective effects of SM (400 mg/kg, p.o.) in an acute liver toxicity model induced by TAA in rats were associated with reduced inflammation (TNF-α) due to decreased oxidative stress, indicated by diminished ROS, MDA and NO levels and increased GSH content, SOD, HO activities and enhanced Nrf2 expression [115]. Similarly, SB was able to suppress abrin (a potent bio-warfare agent)-induced hepatotoxicity as a result of the mitigation of oxidative stress and inhibition of inflammation in mice [116]. Interestingly, SM in combination with chlorogenic acid showed protective effects against hepatotoxicity caused by doxorubicin in rats. This was associated with the effective maintenance of antioxidant enzyme activities, reducing the expression of pro-inflammatory cytokines (TNF-α and IL-1β), decreasing NF-κB levels and apoptosis biomarkers [117]. SM was shown to protect rats against Zn-induced dopaminergic neuronal loss through the suppression of oxidative stress and inflammation. In fact, SM (100 mg/kg body weight; i.p) was reported to mitigate Zn-induced oxidative stress (SOD, GSH, GST and MDA) and inflammation (NF-κB, IL-1β, IL-6, TNF-α) in the rat brain [118].

### 6.3. Ischemia/Reperfusion (I/R) Models

There are many published reports showing the protective effects of SM/SB in kidneys, lungs, the brain and liver in various I/R models. For instance, in various models of renal I/R, SM showed protective action by dose-dependently decreasing oxidative stress, neutrophil infiltration, histological damage and reducing kidney dysfunction [2]. Furthermore, SM treatment (250 mg/kg/day for 8 days) was able to inhibit I/R-induced oxidative stress (SOD and MDA) and inflammation (Il-1β, IL-6, TNF-α and NF-κB) in the lungs of experimental rats [119]. In addition, SM-loaded chitosan nanoparticles were effective in decreasing cerebral inflammation (IL-6 and TNF-α) associated with improved AO defences (SOD, CAT, GPx, GR, GSH) in the I/R rat brain [120]. Finally, in a model of hepatic I/R injury in rats, SB protective action in the liver was related to decreasing inflammation (eNOS and neutrophil infiltration), inhibiting hepatocyte vacuolisation and the degeneration [121] and amelioration of I/R-induced induction of NF-κB and NLRP3 expression as well as inflammatory liver tissue injuries (e.g., neutrophil and macrophage infiltration, cytoplasmic vacuolation, hepatocyte degeneration, etc. [122]). Silybin phytosome (SIBP) treatment was shown to significantly enhance AO defences (increased SOD activity and GSH levels, decreased MDA) simultaneously with decreasing inflammation markers (TNF-α and IL-6) in the hippocampus and cortex of the cerebral ischemia-reperfusion-injured rats [123]. In a recent study with rats, it was demonstrated that intravenous SB has remote anti-inflammatory protective effects in both lungs and kidneys, after hepatic ischemia/reperfusion, as evidenced by the regulation of inflammatory biomarkers, including TNF-α, IL-6 and MCP-1 [124].

### 6.4. Ageing and Exercising Models

The anti-inflammatory actions of SM/SB have been demonstrated in ageing and exercising animal models. For example, in senescence-accelerated mice, SB (100 and 200 mg/kg for 6 weeks) was illustrated to have anti-inflammatory action by downregulating proinflammatory IL-6 as well as inflammation-associated proteins (iNOS and COX-2), leading to decreased microglial activation [125]. This could be an important anti-ageing mechanism of SM. For example, *Caenorhabditis elegans* treated with SM (25 μM or 50 μM) showed an increase in mean lifespan by 10.1% and 24.8%, respectively, in comparison to untreated controls. Moreover, SM treatment improved tolerance to stress and increased the resistance of animals to Alzheimer’s disease development [126]. Of note, SM (20 mg/kg/day i.p, 4 weeks) was demonstrated to enhance the expression of SIRT1 associated with decreased expression of NF-κB, providing anti-inflammatory effects in the hippocampus of old male rats [127]. In addition, SM was indicated to improve muscle recovery and to ameliorate inflammation and damaged tissue in rats subjected to regular exercise training [128]. Finally, a reduction in lung tissue inflammation was evident in rats subjected to 8-week exercise training due to the administration of SM (100 mg [129]). In elderly rats with HFD-induced liver injury, the combination of SM and vitamin C dietary supplementation with exercise was shown to effectively reduce oxidative stress and liver inflammation [130].

### 6.5. Stress-Induced Injuries

It was clearly demonstrated that oxidative stress and increased expression of pro-inflammatory mediators (TNF-α, IL-1β, IL-6 and CCL2) in the liver and serum of mice affected by restraint stress were mitigated by SM (100 mg/kg [131]). Acute respiratory distress syndrome in rats was also characterised by enhanced inflammatory responses, and SM (50, 100 and 200 mg/kg) was shown to reduce lung damage as a result of the downregulation of inflammation markers (TNF-α, IFN-γ and IL-6) simultaneously with the induction of anti-inflammatory IL-10 in broncho-alveolar lavage fluid [132]. The anti-inflammatory protective effects of SB were also observed in a mouse-based chronic unpredictable stress model, as evident from the decrease in stress-induced inflammatory mediators (serum IL-1β, TNF-α) and improved antioxidant defences (GSH and SOD) in stressed animals [133].

Radiation-induced lung injury, inflammation (the number of inflammatory cells in the bronchoalveolar lavage fluid) and fibrosis were found to be significantly reduced by SB treatment (100 mg/day) in experimental mice [134]. Interestingly, UVB-induced skin damage and inflammation were ameliorated by topical application of SB just before UVB-treatment or immediately after UVB exposure (5 days per week for 8 weeks), as evidenced by the suppression of the UVB-induced inflammatory markers (NF-κB, TNF-α, COX2 and iNOS) in the animal skin [135]. In a later study, with a similar model, SB-loaded hydrogel demonstrated anti-inflammatory effects, indicated by a significant reduction in the inflammation mediators’ levels, including IL-22 and TNF-α in the skin [136].

LPS-induced inflammatory responses and oxidative stress are commonly used in various stress models, and SM/SB was illustrated to have protective anti-inflammatory effects. First of all, LPS-induced lung injury and inflammation in a mouse model were significantly inhibited by SB, as indicated by the suppressed recruitment of airway inflammatory cells (macrophages, T cells and neutrophils) associated with the inhibition of NF-κB signalling and a substantial reduction in the production of proinflammatory cytokines (IL-1β and TNF-α) in bronchoalveolar fluid and serum [74]. Secondly, partial inactivation of multiple mitogen-activated protein kinase signalling pathways in the lungs of LPS-treated rats was a result of SM pretreatment [109]. Thirdly, SM treatment (75 mg/kg BW and 150 mg/kg BW) of mice with multiple-organ damage induced by D-galactose/LPS was associated with a significant improvement in liver function (reduced ALT and AST in the serum). The SM treatment was also responsible for improved AO defences and redox balance (SOD, CAT, GSH, GPx, T-AOC activities and expression of SOD1, SOD2, CAT, GPx, IL-10, Nrf2, HO-1, NQO1, Trx and IκB-α). Importantly, a reduction in inflammation, as evidenced by increased IL-10 and IL-12 and reduced IL-6, IL-1β and TNF-α concentrations and expressions, decreased the expression of NF-κB, NLRP3, COX2 and p38, which were major protective effects of SM in the mouse liver [86].

Recent studies have indicated that cigarette smoke has a pro-inflammatory action [137]. For example, SM (25 and 50 mg/kg; [116]) or SB (10 and 20 mg/kg; [115]) was demonstrated to decrease inflammatory cell count and to supress the secretion of pro-inflammatory cytokines (TNF-α, IL-1β and IL-8) in bronchoalveolar lavage fluid of cigarette smoke-treated mice [138,139]. In a similar fashion, SB was able to supress the smoke- and LPS-induced neutrophilic airway inflammation and reduced MPO expression in the mouse lung [140]. Importantly, SM treatment was associated with the amelioration of lung inflammation, simultaneously with an improvement in antioxidant defences, as evidenced by the upregulation of Nrf2 and HO-1 in a model of HCl-induced acute lung injury in rats [141]. Of note, the anti-inflammatory effects of SM + curcumin-loaded albumin nanoparticles coated by chitosan, evidenced by decreased IL-6 in the lung and reduced CRP in serum, were demonstrated in a mouse model of oleic acid-induced lung injury [142]. In a model of mild traumatic brain injury, SM (50 mg/kg/day for 20 days) was reported to reduce inflammatory responses (TNF-α) in the mouse prefrontal cortex [120] and hippocampus [143,144]. There is also evidence demonstrating that SM-loaded gold nanoparticles were able to improve liver function by enhancing antioxidant defences and decreasing oxidative stress, inflammation and fibrosis in a rat model of extrahepatic cholestasis [145].

The anti-inflammatory properties of SM/SB were demonstrated in various stress models, including restraint stress, acute respiratory distress syndrome and a chronic unpredictable stress model. Furthermore, radiation- and UVB-induced tissue damage and inflammation were also ameliorated by SM/SB treatment in various animal models. When LPS and cigarette smoke were used as inflammation-promoting stress factors, SM/SB also demonstrated significant protective actions. In addition, HCl- and oleic acid-induced lung injuries as well as traumatic brain injuries and inflammatory responses were ameliorated by SM/SB treatments. There was a range of other inflammation-associated medical conditions, including benign prostatic hyperplasia, varicocele, dermatitis and peritoneal dialysis, where the anti-inflammatory effects of SM/SB were clearly demonstrated.

### 6.6. Other In Vivo Model Systems and Disease States

There is evidence suggesting that SM could be considered as a useful treatment for inflammation, as evidenced by decreased serum TNF-α in peritoneal dialysis patients [146]. It was emphasized that SM could show protective ant-inflammatory effects in benign prostatic hyperplasia. In fact, SM significantly ameliorated testosterone-induced NF-κB, COX2 and iNOS, leading to the downregulation of inflammation and hyperplasia [147]. Importantly, SM (50 mg/kg, orally) was demonstrated to partially mitigate the varicocele-induced downregulation of the testicular antioxidant defence network (TAC, SOD and GPx) and decreased COX2 and iNOS expression as well as NO content [148]. Atopic dermatitis (redness, swelling and inflammation) was illustrated to be relieved by the silymarin pluronic-lecithin organogel formulation [149]. Similarly, it was revealed that in a mouse model of irritant contact dermatitis, decreased ear oedema and reduced leukocyte infiltration in damaged tissue were observed due to treatment with hydrogel containing SB nanocapsules [150].

The downregulation of NF-κB activity in a mouse asthma model was suggested to be the main molecular mechanisms of protective effects of SB against ovalbumin-induced airway inflammation [151]. Importantly, it seems likely that SB affects Th cell polarization, as evidenced by a downregulation in the secretion of pro-inflammatory Th1 cytokines, simultaneously with the upregulation of anti-inflammatory Th2 cytokines in vitro. This was associated with a dose-dependent inhibition of the production of Th1 cytokines ex vivo [152]. The inhibition of the release of inflammatory mediators (IL-1β, IL-6 and TNF-α) and reduction in sepsis-induced oxidative damage in rat lung tissues were shown to result from SM administration [153]. Two major SM isomers (isosilybin A and silydianin) were reported to have anti-inflammatory properties associated with their ability to reduce eosinophilic infiltration of the lungs, IL-4 and IL-5 levels and gene expression in bronchoalveolar lavage fluid in a Balb/c mouse model of allergic asthma [154]. In IFN-ß-treated multiple sclerosis patients, SM was demonstrated to suppress Th1 proliferating activity associated with the inhibition of T-bet gene expression and IFN-γ production by the immune cells [155]. In an animal model of multiple sclerosis, SB was illustrated to decrease DC activation and to suppress pathogenic Th17 inflammatory cell responses, significantly attenuating inflammation and demyelination of the central nervous system in experimental mice [156]. Importantly, SM treatment was found to mitigate virus-induced increased pro-inflammatory cytokine production and liver inflammation in a mouse model of Mayaro virus infection [157]. SM/SB was shown to be effective in controlling various diabetic complications in several organs, including neuropathy, retinopathy, impaired healing, hepatopathy, cardiomyopathy, nephropathy and osteoporosis [158,159].

The pathological roles of inflammation in the development and progression of diabetes have recently received substantial attention [160]. Furthermore, SM/SB was reported to ameliorate various diabetic, inflammation-related complications in several organs, including neuropathy, retinopathy, impaired healing, hepatopathy, cardiomyopathy, nephropathy and osteoporosis [158,159]. In a model of STZ-induced diabetes in mice, SM was illustrated to improve antioxidant defence mechanisms and to ameliorate inflammation indexes including pancreatic TNF-α and inflammatory cell infiltration in islets [161]. Similarly, in a rat model of STZ-induced diabetic nephropathy, SM (100, 300 and 900 μg/kg) in nanoliposome form was able to mitigate oxidative stress by improving SOD, CAT and GPx activity in the kidneys. Importantly, the STZ-induced increased expression of inflammatory mediators (IL-8, IL-10, TNF-α, IL-6, TGF-β and ICAM-1) in renal tissue was also ameliorated by SM treatment [162]. In a zebrafish model of type 2 diabetes, a treatment with SM combined with metformin was demonstrated to suppress the expression of important inflammatory genes (IL-1β and TNF-α) associated with the amelioration of intestinal inflammation [163]. In an experiment with ApoE−/− mice fed with a high-fat diet, co-administration of SB with Clopidogrel was found to significantly mitigate the inflammation and endothelial dysfunction in aorta roots [164]. In stroke-induced brain ischemia in rats, SB was able to significantly decrease the expression of Bax and NF-κB, simultaneously with the upregulation of Akt, mTOR, HIF-1α and Bcl-2, leading to the mitigation of neurological deficits, reduced infarct volume and suppressed brain oedema [165].

Inflammation is an important hallmark of many neurodegenerative disorders, including Alzheimer’s disease [166,167] and Parkinson’s disease (PD, [168,169]). Therefore, the protective anti-inflammatory properties of SM/SB are of great importance in the development of effective measures to deal with neurodegenerative disorders. For example, in the 1-methyl-4-phenylpyridinium ion (MPP(+))-treated rat model of PD, in vivo animals were intraperitoneally injected with 10, 50 or 100 mg/kg of SB, starting 1 day before MPP(+) injection. It was clearly shown that the highest SB dose (100 mg/kg) was effective in the amelioration of MPP(+)-induced neurotoxicity in the substantia nigra in a dose-dependent manner. This was associated with the mitigation of MPP(+)-induced increased levels of inflammatory markers, including TNF-α, IL-1β and iNOS [170]. In the same fashion, in a mouse model of Alzheimer’s disease, SB encapsulated in macrophage-derived exosomes was able to decrease astrocyte inflammation-mediated neuronal damage as a result of a reduction in the secretion of proinflammatory cytokines (IL-6, IL-1β and TNF-α), with a concomitant downregulation of the protein expression involved in NF-κB signalling [171]. Importantly, SB was effective in suppressing the prothrombin kringle-2-induced production of proinflammatory mediators, including iNOS, IL-1β and TNF-α, in the mouse substantia nigra [172]. The oral administration of SB (100 mg/kg) was demonstrated to protect against MPTP-induced neurotoxicity by reducing the levels of pro-inflammatory cytokines (TNFα, IL-1β and IL-6) in the striatum and substantia nigra and increasing anti-inflammatory cytokine (IL-10 and IL-4) levels in the substantia nigra in mice [173]. The administration of silymarin (200 mg/kg) to aggression-induced mice was indicated to attenuate inflammation (decreased serum IL-6 and TNF-α) and decrease oxidative stress (GSH, CAT and SOD) in the mouse brain [174]. The lower SM dose (100 mg/kg) also improved AO defences in the brain and decreased TNF-α levels in serum but did not affect the IL-6 concentration in serum. The neuroprotective effects of SM against thiamethoxam (TMX)-induced cortical injury in male rats were investigated. Compromised cortical AO defences (decreased activities of SOD, CAT, MDA and reduced expression of Sod, Cat, GPx and Nrf-2) and elevated inflammation (increased concentration of IL-1β and TNF-α as well as enhanced expression of IL-1β, IL-6, iNOS, TNF-α and NF-κB) were observed in TMX-treated rats. Importantly, pre-treatment with SM (150 mg/kg) provided significant neuroprotective effects associated with the amelioration of disturbed AO defences and restoration of inflammatory balance [175]. In 3-nitropropionic acid-induced neurotoxicity in male mice, SM has shown neuroprotective effects associated with attenuating neuroinflammation (TNF-α, IL-17 and IL-1β) and oxidative stress (MDA, SOD, TAC) as well as improving behavioural deficits [176].

Preeclampsia (PE), a leading cause of maternal and foetal mortality worldwide, was demonstrated to be related to inappropriate inflammatory responses, leading to pathophysiological changes [177,178]. Therefore, research related to the anti-inflammatory action of SM/SM in attempts to ameliorate pathologic changes in patients is quickly developing. For instance, increased endogenous activation of NF-κB as well as TNF-α and IL-1β release by PBMC in the preeclampsia-affected group compared with the normotensive pregnant women was confirmed, and SB was illustrated to decrease the levels of NF-κB and the expression of pro-inflammatory cytokines (TNF-α and IL-1β) in preeclamptic women [179]. Furthermore, in patients with preeclampsia, SB was able to suppress the spontaneous and LPS-induced NF-κB activation and production of inflammatory cytokines [179]. In monocytes from preeclamptic pregnant women, SB demonstrated antioxidant and anti-inflammatory activities, as evidenced by the inhibition of pro-inflammatory cytokine (TNF-α) production and the in vitro endogenous release of ROS [180]. Of note, SB (200 μM) was shown to significantly reduce the LPS-induced expression of IL-6 and IL-8, COX-2 and prostaglandins PGE2 and PGF2α in human foetal membranes [181]. Finally, in pregnant preeclamptic women, SB was effective in downregulating the NF-κB pathway, including decreased expression of proinflammatory cytokines (IL-1β, IL-18 and TNF-α) and NLRP1/NLRP3 inflammasomes in monocytes [51,75]. In addition, in pregnant women with preeclampsia, SB (100 μM) was reported to downregulate the expression of the Th1 and Th17 profiles by decreasing inflammatory transcription factors (STAT1/STAT4/T-bet and STAT3/RORγt) and increasing anti-inflammatory (STAT6/GATA-3 and STAT5/FoxP3) transcription factors. This was associated with a reduction in the release of inflammatory cytokines and increased levels of anti-inflammatory IL-10 and TGF-β [182].

Chronic inflammation and oxidative stress are considered to be among the most important factors promoting immunological abnormalities and enhanced susceptibility to infections observed in patients with thalassemia [183]. In fact, thalassemia patients are characterised by enhanced inflammation (decreased expression of anti-inflammatory IL-10 and increased concentrations of pro-inflammatory TGF-β and IL-23), and, in such conditions, SM treatment was demonstrated to have anti-inflammatory effects [184]. Similarly, in beta-thalassemia major patients, there was a redox disbalance, evidenced by a reduction in the GSH concentration, and it was associated with decreased proliferation of PHA-activated PBMC. Importantly, SM treatment was illustrated to effectively restore both GSH levels and PBMC proliferation [185]. More recent observations confirmed the anti-inflammatory actions of SWM/SM in thalassemia patients. For instance, in patients with β-thalassemia major, SM (140 mg, 3 times a day for 12 weeks) was able to improve the inflammatory status, as evidenced by decreased CRP and IL-6 and increased IL-10 levels [186].

In a rat model of L-arginine-induced acute pancreatitis, pretreatment with SB was associated with the amelioration of inflammation, as evidenced by the prevention of histopathological changes in the pancreas and decreased oxidative stress [187]. In *Aspergillus fumigatus-*treated pulmonary microvascular endothelial cells, SB treatment was demonstrated to significantly downregulate the expression of proinflammatory factors and adhesion molecules (IL-6, IL-1β and ICAM-1) simultaneously with the suppression of *A. fumigatus*-induced p38 MAPK activation [188]. Interestingly, in a mouse model of pulmonary fibrosis, SB was indicated to mitigate the alleviated inflammatory markers, including IL-1β, IL-6 and TNF-α, in the fibrotic tissue [189]. Furthermore, in a double-blind clinical trial with patients suffering from painful knee osteoarthritis, SM (300 mg/day for 8 weeks) was found to significantly reduce IL-1α and IL-8 levels and complement proteins C3 and C4 in serum compared to the pre-treatment levels [190].

In a model of experimental gastric ulcer in rats, SM (50 mg/kg orally) pretreatment was reported to suppress gastric inflammation, as evidenced by reduced MPO, TNF-α and IL-6 levels and NF-κB expression. The anti-inflammatory action of SM was associated with an improvement in the AO defences, indicated by the upregulation of Nrf2 and enhancement of SOD and GPx activities [191]. Similarly, in a rat model of experimental colitis, SB was demonstrated to ameliorate intestinal inflammation and decrease colonic tissue injury due to the inhibition of NF-κB activity and neutrophil infiltration/activation in the inflamed colon, simultaneously with a decreased expression of proinflammatory cytokines, such as TNF-α and IL-1β [192]. The protective effects of SM and its nanoemulsion on 5-fluorouracil-induced gastrointestinal toxicity in male rats have been demonstrated [193]. This was evidenced by the decreased gene expression of inflammatory markers (IL-2 and TNF-α), reduced oxidative stress (MDA and TAC) and decreased histopathological degeneration in the gut due to SM treatment. Since intestinal inflammation is a driving force of development of many different diseases/disorders, which cause decreased productive and reproductive performance of poultry and farm animals [194], the possible protective role of SM in the gut warrants further research.

### 6.7. Wound Healing

Accumulating evidence indicates that the effective control of inflammatory reactions is of great importance in wound healing. For example, topical application of SM was reported to be able to improve the biochemical, morphological and biomechanical properties of experimentally induced wound defects in rats [195]. SM topical application was also shown to significantly reduce inflammation and improve epithelialisation in full-thickness wounds in rats [196]. Of note, in patients with second-degree burns, a 4-week adjuvant treatment with oral SM promoted fuller and faster wound recovery [197]. By downregulating phosphorylated NF-κB p65, SM is shown to be involved in the regulation of type I collagen-enhanced migration in murine 3T3-L1 preadipocytes, a vital process in adipocyte tissue wound healing [198]. In a rat model of experimental Achilles tendon, SM treatment led to an improved healing process, as evidenced by a significant improvement in tissue healing indices, the functional index and biomechanical properties [199].

## 7. Silymarin and NF-κB Regulation

The anti-inflammatory properties of phenolic compounds have been demonstrated in a range of in vitro and in vivo studies, and polyphenols may affect inflammation as modulators of inflammatory redox signalling pathways [200]. It is generally accepted that the polyphenolic compounds demonstrate anti-inflammatory activities by modulating the expression of pro-inflammatory genes, including cyclooxygenase, lipoxygenase, nitric oxide synthases and cytokines, mainly through the regulation of NF-κB and MAPK signalling [200,201]. Recent studies have confirmed that the protective anti-inflammatory effects of SM/SB could be mediated by their inhibitory potential on NF-κB, which is a key transcriptional factor for numerous genes involved in the regulation of inflammation, immune system, cell differentiation, survival, apoptosis, etc. [21,23]. Due to the great number of studies devoted to the regulatory roles of SM/SB on NF-κB expression in various in vitro and in vivo model systems, in this paper, we focus only on recent investigations addressing the issue.

### 7.1. In Vitro Studies

SB is a potent inhibitor of NF-κB activation. For example, 25 years ago, Manna et al. [202] tested SB in a number of in vitro human cell experimental systems and found it inhibited TNF-mediated NF-κB activation in a dose-dependent manner, showing that as an NF-κB regulator, SB was 100-times more effective than aspirin. SM was found to be effective in inhibiting T-cell activation and proliferation by inhibiting NF-κB activation/translocation. In particular, in CD4+ splenocytes from C57/Bl6 mice, SM (50 μM) significantly inhibited CD4+ cell proliferation, suppressed IL-2 and IFN-γ production and blocked nuclear translocation of transcription factor NF-κB. Moreover, SM was reported to inhibit p65/NF-κB phosphorylation in CD4+ T cell [203].

It heat-stressed chicken hepatocytes, there was a significant upregulation of oxidative stress biomarkers, including increased MDA and inhibited activities of AO enzymes (SOD, CAT, GR). Furthermore, heat stress was responsible for the increased expression of pro-inflammatory indexes (IL-1β, TNF-α, NF-κB, COX-2 and iNOS), while SM (259 μM) was able to normalise the expression of all those biomarkers [204]. SB was clearly demonstrated to suppress the expression of NF-κB at transcriptional and translational levels in a mouse hepatocyte cell line [205]. In a similar fashion, SB treatment (25 and 50 µM) of LPS-induced human hepatocytes (LO2 cells) was observed to reduce the nuclear translocation of NF-κB and decrease the induction of downstream pro-inflammatory cytokines [206]. In a rat hepatoma cell model of NAFLD progression, based on cell exposure to oleate/palmitate followed by TNF-α treatment, SB (50 µM) was shown to decrease the activation of NF-κB and autophagy turnover [207]. In a dermal inflammatory model based on the LPS-induced hoof dermal cells of dairy cows, SM was illuminated to inhibit the phosphorylation of p65 NF-κB and p38 MAPK associated with decreased secretions of pro-inflammatory cytokines (IL-1β and TNF-α [208]). In a bladder cancer cell line, SB was reported to inhibit the radiotherapy-induced NF-κB and PI3K pathways [209]. In a model system based on murine 3T3-L1 preadipocytes, SB reduced NF-κB p65 activity and decreased ROS generation simultaneously with the induction of sirt1 expression [210]. In an imiquimod-induced psoriasis mouse model, SM was found to suppress inflammatory responses in keratinocytes by inhibiting the transcriptional activity of NF-κB [211]. However, there is a need for further studies on NF-κB inhibition by SB in physiologically relevant (0.2–2 μM) concentrations.

### 7.2. In Vivo Studies

It has been reported that SB (50 mg/kg/BW) has a protective effect against D-galactose-induced senescence as a result of the inhibition of NF-κB activation and ROS production [212]. Furthermore, SB (75 mg/kg/BW) significantly reduced the As-induced overexpression of NF-κB and inhibited caspase-3-mediated tubular cell apoptosis [213]. In a nonalcoholic steatohepatitis mouse model based on a methionine-choline deficient diet, increased NF-κB p65 and p50 binding activities were recorded, while SB (20 mg/kg/BW/i.p.) significantly reduced the activity of both sub-units of NF-κB [214]. Importantly, SM (200 mg/kg/BW) was found to decrease the CCl_4_-induced hepatic NF-κB expression and reduced CCl_4_-induced liver fibrosis [215]. Decreased expression of the NF-κB mRNA and protein in the rat brain due to SB treatment (150 mg/kg/BW/intragastric) was observed in an animal model of cerebral ischemia [216]. In a D-galactosamine-induced hepatotoxicity model in rats, SM treatment (25 mg/kg/BW) was shown to significantly downregulate the expressions of mRNA and protein expression of NF-κB [217]. In cirrhosis rats, SM (50 g/kg) was illustrated to significantly decrease elevated serum levels of NF-κB, IL-6 and transforming growth factor (TGF-β [218]). Furthermore, in alcoholic liver fibrosis in guinea pigs, SM (250 mg/kg/BW) was found to inhibit the NF-κB signalling cascade [219,220]. In a mouse model of nonalcoholic steatohepatitis, SB treatment was reported to suppress the NF-κB-signalling pathway to alleviate the severity of hepatic inflammation and steatohepatitis [221]. Similarly, SB was demonstrated to inhibit NF-κB, leading to the mitigation of renal fibrosis in vitro and in vivo [222]. In insulin-resistant rats, SM (100 mg/kg/day) was indicated to inhibit the AlCl_3_-induced activation of the TLR4 signalling pathway, including the downstream induction of NF-κB [223]. In a rat model of scopolamine-induced dementia, SM pretreatment (200, 400, 800 mg/kg) mitigated the enhanced protein expression of NF-κB in the brain cortex and hippocampus and partially prevented toxicant-induced histopathological changes [224]. Similarly, SM treatment (200 mg/kg) was able to significantly downregulate the inflammatory responses, indicated by the inhibition of NF-κB-p65 levels and preventing NLRP3 inflammasome activation in a mouse model of intracerebral haemorrhages. Importantly, the anti-inflammatory protective action of SM was related to the improved AO defence network, indicated by increased Nrf-2/HO-1 expression [225]. Interestingly, SB (10, 20 and 40 mg/kg) was reported to reduce LPS-induced NF-κB activation and inhibited the expression of the NLRP3 inflammasome, causing the suppression of inflammatory cytokine production in the bronchoalveolar lavage fluid in a mouse model of LPS-induced acute lung injury [226]. In a mouse model of irinotecan-induced nonalcoholic steatohepatitis, SM (1.5 mg/kg) was demonstrated to mitigate increased IL-1β and IL-6 levels and prevented NF-κB overexpression in the liver [227]. Recently, it has been demonstrated that SB is able to mitigate STING-mediated neuroinflammation via the downregulation of ferroptotic damage [228]. Similarly, SM was reported to ameliorate the phosphorylation of NF-κB p65 and p38 MAP kinase, inhibited STZ-induced overexpression of inflammatory cytokines, vascular endothelial growth factor (VEGF), adhesion molecules and extracellular matrix proteins in rats [229].

**Figure 3:**
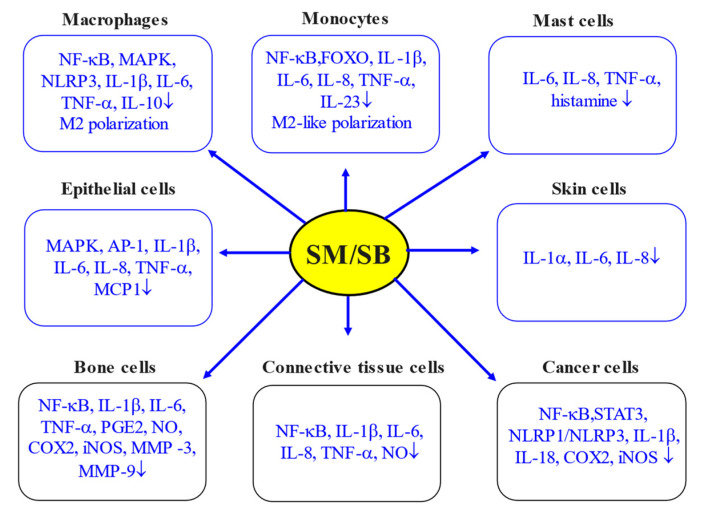
Anti-inflammatory action of SM/SB in various in vitro systems (adapted from [2]). Arrows indicate cell types and inflammatory parameters affected by SM/SB.

Therefore, SM/SB, as one of the most widely studied phytochemicals with antioxidant and anti-inflammatory properties [2,5,6], has received significant attention for the last 30 years. The aforementioned research data clearly demonstrated that SM/SB has great potential as an anti-inflammation agent (Figure 3). In particular, in in vitro studies, SM/SB was illustrated to downregulate NF-κB and downstream proinflammatory cytokines and chemokines, including TNF-α, IL-1β, IL-6, IL-12, IL-23, CCL4 and CXCL10.

Of note, in the same model systems, SM/SB was able to upregulate anti-inflammatory cytokines (IL-4, IL-10, IL-13, TGF-β, etc.) and lipid mediators involved in the resolution of inflammation. The inflammatory properties of SM/SB were clearly shown in model systems based on immune (macrophages and monocytes) and non-immune (epithelial, skin, bone, connective tissue and cancer) cells. At the same time, the anti-inflammatory action of SM/SB was clearly confirmed in several in vivo models, including toxicity models, nonalcoholic fatty liver disease, ischemia/reperfusion models, stress-induced injuries, ageing and exercising models, wound healing and many other relevant model systems.

Overall, it is clearly shown that SM/SB targets multiple signalling pathways, including NF-κB, leading to the inhibition of the secretion of pro-inflammatory cytokines and preventing over-inflammation. It seems likely that targeting the NF-κB signalling pathway by SM/SB may have potential practical applications in the clinical sciences and veterinary medicine.

## 8. Conclusions

Inflammation is generally known to be an important immunological defence mechanism, responsible for the creation of hostile conditions for penetrating pathogens, restricting the spread of tissue infection and maintaining cell/tissue homeostasis. However, as a result of various stresses and redox balance alterations, inflammation can be misregulated, leading to damage to various tissues and organs [2,4]. The consequences of oxidative stress and over-inflammation are known to cause various human health complications, including obesity, metabolic syndrome, type 2 diabetes, atherosclerosis and other cardiovascular diseases, chronic kidney disease as well as auto-immune diseases [230]. Similarly, low-grade chronic inflammation in poultry/animal production systems [231] is also of great importance for health maintenance and for achieving high productive and reproductive performance [13,232,233,234].

Pro-inflammatory stress includes a number of universal components, as follows [2,4,235]:

- Oxidative stress response;
- DNA damage response;
- Unfolded protein response;
- Heat shock response;
- Autophagy modulation;
- Formation of inflammasome;
- Non-coding RNA response;
- Inducible networks of signalling pathway formation and epigenetic changes.

When the binding energies of SM with four different inflammation-related compounds, e.g., NF-κB, MAP kinase, COX-2 and PLA2, were calculated, they were shown to be in a range from −5.29 to −9.18 kcal/mol. Therefore, the docking data indicated that SM could act as a potent anti-inflammatory molecule because of negative binding energies and hydrogen bonding [236]. Accumulating evidence indicates that SM/SB possesses anti-inflammatory properties, demonstrated in both in vitro and in vivo inflammatory disease models, being promising candidates for the prophylactic/treatment of several inflammatory diseases. The main anti-inflammatory mechanism of SM/SB action is attributed to an inhibition of TLR4/NF-κB-mediated signalling pathways and the downregulated expression of pro-inflammatory mediators [2,237]. A recent phenotypic screen identified SM as a novel anti-inflammatory analgesic [238]. When the authors screened a library of 906 bioactive compounds, they discovered 24 hits that decreased calcium flux elicited by agonists. Among those hits, SM was found to significantly supress activation by the stimulation cocktail, as well as by a mixture of bradykinin and prostaglandin E2. In addition, in an in vivo study, SM pretreatment was able to block the development of adjuvant-mediated thermal hypersensitivity [238].

Furthermore, SM showed promising results as a preventive or therapeutic measure for chemotherapy and radiotherapy-induced adverse reactions, including inflammation [239]. For example, SM was reported to protect the liver from the harmful effects of the anticancer drug paclitaxel, showing anti-inflammatory (TNF-α), anti-apoptotic and antioxidant effects [240]. Therefore, it could be concluded that the anti-inflammatory properties of SM/SB are key elements of health-promoting properties of these phytochemicals, and further research should pay more attention to the molecular mechanisms of the protective actions of this phytochemical.

## Figures and Tables

**Table 1:** Inflammatory factors in humans and poultry (adapted from [2,3,4]).

Category	Proinflammatory Factors	
	Human	Poultry
Physical factors	Radiation, UV, hyperthermia, hypothermia, trauma	Hyperthermia, hypothermia, trauma, increased stocking density
Chemical factors	Asbestos, heavy metals, organic toxicants, dust, lipopolysaccharides	Heavy metals, mycotoxins, ammonia, CO, dust
Biological factors	Bacterial infection, viral infection, fungal infection	Bacterial infection, viral infection, fungal infection
Unhealthy lifestyle	Smoking, alcohol, high-calorie diet, stress, sedentary lifestyle	Restricted movement (cage housing), nutrient deficiency
Chronic diseases	Obesity, diabetes, hyperglycaemia	Chronic Respiratory Disease

## References

[1] Lei Y., Wang K., Deng L., Chen Y., Nice E.C., Huang C. (2015). Redox regulation of inflammation: Old elements, a new story. Med. Res. Rev..

[2] Surai P.F., Surai A., Surai P.F., Surai A. (2023). Silymarin and inflammation: From understanding molecular mechanisms to practical applications. Silymarin Puzzle.

[3] Yu W., Tu Y., Long Z., Liu J., Kong D., Peng J., Wu H., Zheng G., Zhao J., Chen Y. (2022). Reactive Oxygen Species Bridge the Gap between Chronic Inflammation and Tumor Development. Oxid. Med. Cell Longev..

[4] Surai P.F. (2020). Vitagenes in Avian Biology and Poultry Health.

[5] Surai P.F. (2015). Silymarin as a natural antioxidant: An overview of the current evidence and perspectives. Antioxidants.

[6] Surai P.F., Ostojic S.M. (2023). Silymarin as a vitagene modulator: Effects on mitochondria integrity in stress conditions. Molecular Nutrition and Mitochondria.

[7] Sharma S., Kumar P., Ashawat M.S., Pandit V., Verma C.S., Sharma D.K. (2023). Silymarin: A Phytoconstituent with Significant Therapeutic Potential-A Narrative Review. Curr. Drug Ther..

[8] Pérez S., Rius-Pérez S. (2022). Macrophage Polarization and Reprogramming in Acute Inflammation: A Redox Perspective. Antioxidants.

[9] Chen L., Deng H., Cui H., Fang J., Zuo Z., Deng J., Li Y., Wang X., Zhao L. (2018). Inflammatory responses and inflammation-associated diseases in organs. Oncotarget.

[10] Schmid-Schönbein G.W. (2006). Analysis of inflammation. Annu. Rev. Biomed. Eng..

[11] Ahmed A.U. (2011). An overview of inflammation: Mechanism and consequences. Front. Biol..

[12] Liu D., Zhong Z., Karin M. (2022). NF-κB: A double-edged sword controlling inflammation. Biomedicines.

[13] Broom L.J., Kogut M.H. (2018). Inflammation: Friend or foe for animal production?. Poult. Sci..

[14] Robinson M.W., Harmon C., O’Farrelly C. (2016). Liver immunology and its role in inflammation and homeostasis. Cell. Mol. Immunol..

[15] Jain P., Pandey R., Shukla S.S. (2015). Inflammation: Natural Resources and Its Applications.

[16] Fernandes Q., Inchakalody V.P., Bedhiafi T., Mestiri S., Taib N., Uddin S., Merhi M., Dermime S. (2023). Chronic inflammation and cancer; the two sides of a coin. Life Sci..

[17] Surai P.F., Surai A., Surai P.F., Surai A. (2023). Antioxidant defence systems in health and diseases. Silymarin Puzzle.

[18] Sies H., Jones D.P. (2020). Reactive oxygen species (ROS) as pleiotropic physiological signalling agents. Nat. Rev. Mol. Cell Biol..

[19] Bhattacharya S., Rattan S.I., Rattan S.I.S., Kyriazis M. (2019). Primary stress response pathways for preconditioning and physiological hormesis. The Science of Hormesis in Health and Longevity.

[20] Surai P.F., Kochish I.I., Fisinin V.I., Kidd M.T. (2019). Antioxidant Defence Systems and Oxidative Stress in Poultry Biology: An Update. Antioxidants.

[21] Surai P.F., Surai A., Surai P.F., Surai A. (2023). Silymarin and Nrf2. Silymarin Puzzle.

[22] Surai P.F., Kochish I.I., Kidd M.T. (2021). Redox Homeostasis in Poultry: Regulatory Roles of NF-κB. Antioxidants.

[23] Surai P.F., Surai A., Surai P.F., Surai A. (2023). Silymarin and NF-κB. Silymarin Puzzle.

[24] Austermann J., Roth J., Barczyk-Kahlert K. (2022). The good and the bad: Monocytes’ and macrophages’ diverse functions in inflammation. Cells.

[25] Juráňová J., Aury-Landas J., Boumediene K., Baugé C., Biedermann D., Ulrichová J., Franková J. (2019). Modulation of skin inflammatory response by active components of silymarin. Molecules.

[26] Bijak M. (2017). Silybin, a major bioactive component of milk thistle (*Silybum marianum* L. Gaernt.)—Chemistry, bioavailability, and metabolism. Molecules.

[27] Křen V., Valentová K. (2022). Silybin and its congeners: From traditional medicine to molecular effects. Nat. Prod. Rep..

[28] Tvrdý V., Pourová J., Jirkovský E., Křen V., Valentová K., Mladěnka P. (2021). Systematic review of pharmacokinetics and potential pharmacokinetic interactions of flavonolignans from silymarin. Med. Res. Rev..

[29] Wang X., Zhang Z., Wu S.C. (2020). Health benefits of *Silybum marianum*: Phytochemistry, pharmacology, and applications. J. Agric. Food Chem..

[30] Aziz M., Saeed F., Ahmad N., Ahmad A., Afzaal M., Hussain S., Mohamed A.A., Alamri M.S., Anjum F.M. (2020). Biochemical profile of milk thistle (*Silybum marianum* L.) with special reference to silymarin content. Food Sci. Nutr..

[31] Martinelli T., Fulvio F., Pietrella M., Focacci M., Lauria M., Paris R. (2021). In *Silybum marianum* Italian wild populations the variability of silymarin profiles results from the combination of only two stable chemotypes. Fitoterapia.

[32] Devi K.P., Seyed N., Silva A. (2019). Milk thistle (*Silybum marianum*). Nonvitamin and Nonmineral Nutritional Supplements.

[33] Abenavoli L., Izzo A.A., Milić N., Cicala C., Santini A., Capasso R. (2018). Milk thistle (*Silybum marianum*): A concise overview on its chemistry, pharmacological, and nutraceutical uses in liver diseases. Phytother. Res..

[34] Fallah M., Davoodvandi A., Nikmanzar S., Aghili S., Mirazimi S., Aschner M., Rashidian A., Hamblin M.R., Chamanara M., Naghsh N. (2021). Silymarin (milk thistle extract) as a therapeutic agent in gastrointestinal cancer. Biomed. Pharmacother..

[35] Křen V. (2021). Chirality matters: Biological activity of optically pure silybin and Its congeners. Int. J. Mol. Sci..

[36] Kim N.C., Graf T.N., Sparacino C.M., Wani M.C., Wall M.E. (2003). Complete isolation and characterization of silybins and isosilybins from milk thistle (*Silybum marianum*). Org. Biomol. Chem..

[37] Degboé Y., Poupot R., Poupot M. (2022). Repolarization of unbalanced macrophages: Unmet medical need in chronic inflammation and cancer. Int. J. Mol. Sci..

[38] Rendra E., Riabov V., Mossel D.M., Sevastyanova T., Harmsen M.C., Kzhyshkowska J. (2019). Reactive oxygen species (ROS) in macrophage activation and function in diabetes. Immunobiology.

[39] Kapellos T.S., Iqbal A.J. (2016). Epigenetic control of macrophage polarisation and soluble mediator gene expression during inflammation. Mediat. Inflamm..

[40] Kim E.J., Lee M.Y., Jeon Y.J. (2015). Silymarin inhibits morphological changes in LPS-stimulated macrophages by blocking NF-κB pathway. Korean J. Physiol. Pharmacol..

[41] Lu C.P., Huang C.Y., Wang S.H., Chiu C.H., Li L.H., Hua K.F., Wu T.H. (2018). Improvement of hyperglycemia in a murine model of insulin resistance and high glucose- and inflammasome-mediated IL-1β expressions in macrophages by silymarin. Chem. Biol. Interact..

[42] Chen J., Li D.L., Xie L.N., Ma Y.R., Wu P.P., Li C., Liu W.F., Zhang K., Zhou R.P., Xu X.T. (2020). Synergistic anti-inflammatory effects of silibinin and thymol combination on LPS-induced RAW264.7 cells by inhibition of NF-κB and MAPK activation. Phytomedicine.

[43] Zheng Y., Chen J., Wu X., Zhang X., Hu C., Kang Y., Lin J., Li J., Huang Y., Zhang X. (2022). Enhanced anti-inflammatory effects of silibinin and capsaicin combination in lipopolysaccharide-induced RAW264.7 cells by inhibiting NF-κB and MAPK activation. Front. Chem..

[44] Huang R.Y., Chang H.Y., Chih S.M., Dyke T.V., Cheng C.D., Sung C.E., Weng P.W., Shieh Y.S., Cheng W.C. (2023). Silibinin alleviates inflammation-induced bone loss by modulating biological interaction between human gingival fibroblasts and monocytes. J. Periodontol..

[45] Mi X.J., Le H.M., Lee S., Park H.R., Kim Y.J. (2022). Silymarin-Functionalized Selenium Nanoparticles Prevent LPS-Induced Inflammatory Response in RAW264.7 Cells through Downregulation of the PI3K/Akt/NF-κB Pathway. ACS Omega.

[46] Liu Z., Sun M., Wang Y., Zhang L., Zhao H., Zhao M. (2018). Silymarin attenuated paraquat-induced cytotoxicity in macrophage by regulating Trx/TXNIP complex, inhibiting NLRP3 inflammasome activation and apoptosis. Toxicol. Vitr..

[47] Zhang B., Xu D., She L., Wang Z., Yang N., Sun R., Zhang Y., Yan C., Wei Q., Aa J. (2018). Silybin inhibits NLRP3 inflammasome assembly through the NAD^+^/SIRT2 pathway in mice with nonalcoholic fatty liver disease. FASEB J..

[48] Bittencourt M., Rodrigues R.P., Kitagawa R.R., Gonçalves R. (2020). The gastroprotective potential of silibinin against Helicobacter pylori infection and gastric tumor cells. Life Sci..

[49] Azadpour M., Farajollahi M.M., Dariushnejad H., Varzi A.M., Varezardi A., Barati M. (2021). Effects of synthetic silymarin-PLGA nanoparticles on M2 polarization and inflammatory cytokines in LPS-treated murine peritoneal macrophages. Iran. J. Basic Med. Sci..

[50] Xu W., Sun Y., Wang J., Wang B., Xu F., Xie Z., Wang Y. (2022). Controlled release of silibinin in GelMA hydrogels inhibits inflammation by inducing M2-type macrophage polarization and promotes vascularization in vitro. RSC Adv..

[51] Bannwart C.F., Nakaira-Takahagi E., Golim M.A., de Medeiros L.T., Romão M., Weel I.C., Peraçoli M.T. (2010). Downregulation of nuclear factor-kappa B (NF-kappaB) pathway by silibinin in human monocytes challenged with Paracoccidioides brasiliensis. Life Sci..

[52] Giorgi V.S., Bannwart-Castro C.F., Peracoli J.C., Peracoli M.T. (2012). Silibinin modulates NF-κB pathway and proinflammatory cytokines production by mononuclear cells of preeclamptic women. Pregnancy Hypertens..

[53] Kim B.R., Seo H.S., Ku J.M., Kim G.J., Jeon C.Y., Park J.H., Jang B.H., Park S.J., Shin Y.C., Ko S.G. (2013). Silibinin inhibits the production of pro-inflammatory cytokines through inhibition of NF-κB signaling pathway in HMC-1 human mast cells. Inflamm. Res..

[54] Choi Y.H., Yan G.H. (2009). Silibinin attenuates mast cell-mediated anaphylaxis-like reactions. Biol. Pharm. Bull..

[55] Fordham J.B., Naqvi A.R., Nares S. (2014). Leukocyte production of inflammatory mediators is inhibited by the antioxidants phloretin, silymarin, hesperetin, and resveratrol. Mediat. Inflamm..

[56] Lovelace E.S., Wagoner J., MacDonald J., Bammler T., Bruckner J., Brownell J., Beyer R.P., Zink E.M., Kim Y.M., Kyle J.E. (2015). Silymarin suppresses cellular inflammation by inducing reparative stress signaling. J. Nat. Prod..

[57] Lovelace E.S., Maurice N.J., Miller H.W., Slichter C.K., Harrington R., Magaret A., Prlic M., De Rosa S., Polyak S.J. (2017). Silymarin suppresses basal and stimulus-induced activation, exhaustion, differentiation, and inflammatory markers in primary human immune cells. PLoS ONE.

[58] Gugliandolo E., Crupi R., Biondi V., Licata P., Cuzzocrea S., Passantino A. (2020). Protective effect of silibinin on lipopolysaccharide-induced inflammatory responses in equine peripheral blood mononuclear cells, an in vitro study. Animals.

[59] Gomes V.J., Nunes P.R., Matias M.L., Ribeiro V.R., Devides A.C., Bannwart-Castro C.F., Romagnoli G.G., Peraçoli J.C., Peraçoli M., Romao-Veiga M. (2020). Silibinin induces in vitro M2-like phenotype polarization in monocytes from preeclamptic women. Int. Immunopharmacol..

[60] Dupuis M.L., Conti F., Maselli A., Pagano M.T., Ruggieri A., Anticoli S., Fragale A., Gabriele L., Gagliardi M.C., Sanchez M. (2018). The natural agonist of estrogen receptor β Silibinin plays an immunosuppressive role representing a potential therapeutic tool in rheumatoid arthritis. Front. Immunol..

[61] Li D., Hu J., Wang T., Zhang X., Liu L., Wang H., Wu Y., Xu D., Wen F. (2016). Silymarin attenuates cigarette smoke extract-induced inflammation via simultaneous inhibition of autophagy and ERK/p38 MAPK pathway in human bronchial epithelial cells. Sci. Rep..

[62] Miethe C., Nix H., Martin R., Hernandez A.R., Price R.S. (2017). Silibinin reduces the impact of obesity on invasive liver cancer. Nutr. Cancer.

[63] Lim R., Morwood C.J., Lim J.O., Shin N.R., Seo Y.S., Nam H.H., Ko J.W., Jung T.Y., Lee S.J., Kim H.J. (2020). Silibinin attenuates silica dioxide nanoparticles-induced inflammation by suppressing TXNIP/MAPKs/AP-1 signaling. Cells.

[64] Speciale A., Muscarà C., Molonia M.S., Cimino F., Saija A., Giofrè S.V. (2021). Silibinin as potential tool against SARS-CoV-2: In silico spike receptor-binding domain and main protease molecular docking analysis, and in vitro endothelial protective effects. Phytother. Res..

[65] Xu S., Jiang X., Liu Y., Jiang X., Che L., Lin Y., Zhuo Y., Feng B., Fang Z., Hua L. (2023). Silibinin Alleviates Lipopolysaccharide Induced Inflammation in Porcine Mammary Epithelial Cells via mTOR/NF-κB Signaling Pathway. Mol. Nutr. Food Res..

[66] Tewari-Singh N., Jain A.K., Inturi S., Agarwal C., White C.W., Agarwal R. (2012). Silibinin attenuates sulfur mustard analog-induced skin injury by targeting multiple pathways connecting oxidative stress and inflammation. PLoS ONE.

[67] Frankova J., Juranova J., Biedermann D., Ulrichova J. (2021). Influence of silymarin components on keratinocytes and 3D reconstructed epidermis. Toxicol. Vitr..

[68] Zheng W., Feng Z., Lou Y., Chen C., Zhang C., Tao Z., Li H., Cheng L., Ying X. (2017). Silibinin protects against osteoarthritis through inhibiting the inflammatory response and cartilage matrix degradation in vitro and in vivo. Oncotarget.

[69] Fernandes C., Veiga M.R., Peracoli M., Zambuzzi W.F. (2019). Modulatory effects of silibinin in cell behavior during osteogenic phenotype. J. Cell. Biochem..

[70] Tong W.W., Zhang C., Hong T., Liu D.H., Wang C., Li J., He X.K., Xu W.D. (2018). Silibinin alleviates inflammation and induces apoptosis in human rheumatoid arthritis fibroblast-like synoviocytes and has a therapeutic effect on arthritis in rats. Sci. Rep..

[71] Sharifi R., Pasalar P., Kamalinejad M., Dehpour A.R., Tavangar S.M., Paknejad M., Mehrabani Natanzi M., Nourbakhsh M., Ahmadi Ashtiani H.R., Akbari M. (2013). The effect of silymarin (*Silybum marianum*) on human skin fibroblasts in an in vitro wound healing model. Pharm. Biol..

[72] Dobiasová S., Řehořová K., Kučerová D., Biedermann D., Káňová K., Petrásková L., Koucká K., Václavíková R., Valentová K., Ruml T. (2020). Multidrug resistance modulation activity of silybin derivatives and their anti-inflammatory Potential. Antioxidants.

[73] Raina K., Agarwal C., Agarwal R. (2013). Effect of silibinin in human colorectal cancer cells: Targeting the activation of NF-κB signaling. Mol. Carcinog..

[74] Zhang B., Wang B., Cao S., Wang Y., Wu D. (2017). Silybin attenuates LPS-induced lung injury in mice by inhibiting NF-κB signaling and NLRP3 activation. Int. J. Mol. Med..

[75] Matias M.L., Gomes V.J., Romao-Veiga M., Ribeiro V.R., Nunes P.R., Romagnoli G.G., Peracoli J.C., Peracoli M. (2019). Silibinin downregulates the NF-κB pathway and NLRP1/NLRP3 inflammasomes in monocytes from pregnant women with preeclampsia. Molecules.

[76] Cho K., Lee H.G., Piao J.Y., Kim S.J., Na H.K., Surh Y.J. (2021). Protective effects of silibinin on Helicobacter pylori-induced gastritis: NF-κB and STAT3 as potential targets. J. Cancer Prev..

[77] Faixová D., Ratvaj M., Maruščáková I.C., Hrčková G., Karaffová V., Faixová Z., Mudroňová D. (2023). Silybin Showed Higher Cytotoxic, Antiproliferative, and Anti-Inflammatory Activities in the CaCo Cancer Cell Line while Retaining Viability and Proliferation in Normal Intestinal IPEC-1 Cells. Life.

[78] Yan L., Zhou J., Yuan L., Ye J., Zhao X., Ren G., Chen H. (2023). Silibinin alleviates intestinal inflammation via inhibiting JNK signaling in Drosophila. Front. Pharmacol..

[79] Yassin N., AbouZid S.F., El-Kalaawy A.M., Ali T.M., Elesawy B.H., Ahmed O.M. (2021). Tackling of renal carcinogenesis in Wistar rats by *Silybum marianum* total extract, silymarin, and silibinin via modulation of oxidative stress, apoptosis, Nrf2, PPARγ, NF-κB, and PI3K/Akt signaling pathways. Oxid. Med. Cell. Longev..

[80] Shen Y., Zhao H., Wang Z., Guan W., Kang X., Tai X., Sun Y. (2019). Silibinin declines blue light-induced apoptosis and inflammation through MEK/ERK/CREB of retinal ganglion cells. Artif. Cells Nanomed. Biotechnol..

[81] Chen Y.H., Lin H., Wang Q., Hou J.W., Mao Z.J., Li Y.G. (2020). Protective role of silibinin against myocardial ischemia/reperfusion injury-induced cardiac dysfunction. Int. J. Biol. Sci..

[82] Meng D., Wang Y., Liu T. (2022). Protective effects of silibinin on LPS-induced inflammation in human periodontal ligament cells. Front. Chem..

[83] Li X., Zhou R., Han Y., Zeng J., Shi L., Mao Y., Sun X., Ji Y., Zhang X., Chen Y. (2023). Silibinin Attenuates Experimental Periodontitis by Downregulation of Inflammation and Oxidative Stress. Oxid. Med. Cell. Longev..

[84] Saliou C., Valacchi G., Rimbach G. (2001). Assessing bioflavonoids as regulators of NF-kappa B activity and inflammatory gene expression in mammalian cells. Methods Enzymol..

[85] Podder B., Kim Y.S., Zerin T., Song H.Y. (2012). Antioxidant effect of silymarin on paraquat-induced human lung adenocarcinoma A549 cell line. Food Chem. Toxicol..

[86] Zhao X., Wang H., Yang Y., Gou Y., Wang Z., Yang D., Li C. (2021). Protective effects of silymarin against D-Gal/LPS-induced organ damage and Inflammation in mice. Drug Des. Devel. Ther..

[87] Schümann J., Prockl J., Kiemer A.K., Vollmar A.M., Bang R., Tiegs G. (2003). Silibinin protects mice from T cell-dependent liver injury. J. Hepatol..

[88] Thibaut R., Gage M.C., Pineda-Torra I., Chabrier G., Venteclef N., Alzaid F. (2022). Liver macrophages and inflammation in physiology and physiopathology of non-alcoholic fatty liver disease. FEBS J..

[89] Itoh M., Ogawa Y., Suganami T. (2020). Chronic inflammation as a molecular basis of nonalcoholic steatohepatitis: Role of macrophages and fibroblasts in the liver. Nagoya J. Med. Sci..

[90] Salamone F., Galvano F., Marino A., Paternostro C., Tibullo D., Bucchieri F., Mangiameli A., Parola M., Bugianesi E., Volti G.L. (2012). Silibinin improves hepatic and myocardial injury in mice with nonalcoholic steatohepatitis. Dig. Liver Dis..

[91] Marin V., Gazzin S., Gambaro S.E., Dal Ben M., Calligaris S., Anese M., Raseni A., Avellini C., Giraudi P.J., Tiribelli C. (2017). Effects of oral administration of silymarin in a juvenile murine model of non-alcoholic steatohepatitis. Nutrients.

[92] Lee S.J., Nam M.J., Lee D.E., Park J.W., Kang B.S., Lee D.S., Lee H.S., Kwon O.S. (2018). Silibinin ameliorates O-GlcNAcylation and inflammation in a mouse model of nonalcoholic steatohepatitis. Int. J. Mol. Sci..

[93] Ou Q., Weng Y., Wang S., Zhao Y., Zhang F., Zhou J., Wu X. (2018). Silybin alleviates hepatic steatosis and fibrosis in NASH mice by inhibiting oxidative stress and involvement with the Nf-κB pathway. Dig. Dis. Sci..

[94] Zhang R., Xu D., Zhang Y., Wang R., Yang N., Lou Y., Zhao H., Aa J., Wang G., Xie Y. (2021). Silybin restored CYP3A expression through the Sirtuin 2/Nuclear Factor κ-B pathway in mouse nonalcoholic fatty liver disease. Drug Metab. Dispos..

[95] Gu M., Zhao P., Huang J., Zhao Y., Wang Y., Li Y., Li Y., Fan S., Ma Y.M., Tong Q. (2016). Silymarin ameliorates metabolic dysfunction associated with diet-Induced obesity via activation of Farnesyl X receptor. Front. Pharmacol..

[96] Guo Y., Wang S., Wang Y., Zhu T. (2016). Silymarin improved diet-induced liver damage and insulin resistance by decreasing inflammation in mice. Pharm. Biol..

[97] Alsaggar M., Bdour S., Ababneh Q., El-Elimat T., Qinna N., Alzoubi K.H. (2020). Silibinin attenuates adipose tissue inflammation and reverses obesity and its complications in diet-induced obesity model in mice. BMC Pharmacol. Toxicol..

[98] Shen H.H., Alex R., Bellner L., Raffaele M., Licari M., Vanella L., Stec D.E., Abraham N.G. (2020). Milk thistle seed cold press oil attenuates markers of the metabolic syndrome in a mouse model of dietary-induced obesity. J. Food Biochem..

[99] Li S., Duan F., Li S., Lu B. (2023). Administration of Silymarin in NAFLD/NASH: A systematic review and meta-analysis. Ann. Hepatol..

[100] Luster M.I., Simeonova P.P., Gallucci R.M., Matheson J.M., Yucesoy B. (2000). Immunotoxicology: Role of inflammation in chemical-induced hepatoto-xicity. Int. J. Immunopharmacol..

[101] Clichici S., Olteanu D., Filip A., Nagy A.L., Oros A., Mircea P.A. (2016). Beneficial effects of silymarin after the discontinuation of CCl4-induced liver fibrosis. J. Med. Food.

[102] Sokar S.S., El-Sayad M.E., Ghoneim M.E., Shebl A.M. (2017). Combination of Sitagliptin and silymarin ameliorates liver fibrosis induced by carbon tetrachloride in rats. Biomed. Pharmacother..

[103] Al-Rasheed N., Faddah L., Al-Rasheed N., Bassiouni Y.A., Hasan I.H., Mahmoud A.M., Mohamad R.A., Yacoub H.I. (2016). Protective effects of silymarin, alone or in combination with chlorogenic acid and/or melatonin, against carbon tetrachloride-induced hepatotoxicity. Pharmacogn. Mag..

[104] Zhao X.A., Chen G.M., Liu Y., Chen Y.X., Wu H.Y., Chen J., Xiong Y.L., Tian C., Wang G.Y., Jia B. (2017). Inhibitory effect of silymarin on CCl_4_-induced liver fibrosis by reducing Ly6C^hi^ monocytes infiltration. Int. J. Clin. Exp. Pathol..

[105] El-Kot S.M., Wanas W., Hafez A.M., Mahmoud N.A., Tolba A.M., Younis A.H., Sayed G.E., Abdelwahab H.E. (2023). Effect of silymarin on the relative gene expressions of some inflammatory cytokines in the liver of CCl_4_-intoxicated male rats. Sci. Rep..

[106] Ye Z., Zhang X., Huang Q., Zhang W., Ye M. (2021). Synergistic hepatoprotective effect of combined administration of Lachnum polysaccharide with silymarin. Bioorg. Med. Chem. Lett..

[107] Ahmad I., Shukla S., Kumar A., Singh B.K., Kumar V., Chauhan A.K., Singh D., Pandey H.P., Singh C. (2013). Biochemical and molecular mechanisms of N-acetyl cysteine and silymarin-mediated protection against maneb- and paraquat-induced hepatotoxicity in rats. Chem. Biol. Interact..

[108] Razavi-Azarkhiavi K., Ali-Omrani M., Solgi R., Bagheri P., Haji-Noormohammadi M., Amani N., Sepand M.R. (2014). Silymarin alleviates bleomycin-induced pulmonary toxicity and lipid peroxidation in mice. Pharm. Biol..

[109] Liu W., Li Y., Zheng X., Zhang K., Du Z. (2015). Potent inhibitory effect of silibinin from milk thistle on skin inflammation stimuli by 12-O-tetradecanoylphorbol-13-acetate. Food Funct..

[110] Zaulet M., Kevorkian S., Dinescu S., Cotoraci C., Suciu M., Herman H., Buburuzan L., Badulescu L., Ardelean A., Hermenean A. (2017). Protective effects of silymarin against bisphenol A-induced hepatotoxicity in mouse liver. Exp. Ther. Med..

[111] Wang L., Huang Q.H., Li Y.X., Huang Y.F., Xie J.H., Xu L.Q., Dou Y.X., Su Z.R., Zeng H.F., Chen J.N. (2018). Protective effects of silymarin on triptolide-induced acute hepatotoxicity in rats. Mol. Med. Rep..

[112] Papackova Z., Heczkova M., Dankova H., Sticova E., Lodererova A., Bartonova L., Poruba M., Cahova M. (2018). Silymarin prevents acetaminophen-induced hepatotoxicity in mice. PLoS ONE.

[113] Vangaveti S., Das P., Kumar V.L. (2021). Metformin and silymarin afford protection in cyclosporine A induced hepatorenal toxicity in rat by modulating redox status and inflammation. J. Biochem. Mol. Toxicol..

[114] Yardım A., Kucukler S., Özdemir S., Çomaklı S., Caglayan C., Kandemir F.M., Çelik H. (2021). Silymarin alleviates docetaxel-induced central and peripheral neurotoxicity by reducing oxidative stress, inflammation and apoptosis in rats. Gene.

[115] Hussein R.M., Sawy D.M., Kandeil M.A., Farghaly H.S. (2021). Chlorogenic acid, quercetin, coenzyme Q10 and silymarin modulate Keap1-Nrf2/heme oxygenase-1 signaling in thioacetamide-induced acute liver toxicity. Life Sci..

[116] Saxena N., Dhaked R.K., Nagar D.P. (2022). Silibinin ameliorates abrin induced hepatotoxicity by attenuating oxidative stress, inflammation and inhibiting Fas pathway. Environ. Toxicol. Pharmacol..

[117] Abbas N., Awad M.M., Nafea O.E. (2020). Silymarin in combination with chlorogenic acid protects against hepatotoxicity induced by doxorubicin in rats: Possible role of adenosine monophosphate-activated protein kinase pathway. Toxicol. Res..

[118] Singh G., Mittra N., Singh C. (2023). Tempol and silymarin rescue from zinc-induced degeneration of dopaminergic neurons through modulation of oxidative stress and inflammation. Mol. Cell. Biochem..

[119] Jin Y., Zhao X., Zhang H., Li Q., Lu G., Zhao X. (2016). Modulatory effect of silymarin on pulmonary vascular dysfunction through HIF-1α-iNOS following rat lung ischemia-reperfusion injury. Exp. Ther. Med..

[120] Moghaddam A.H., Mokhtari Sangdehi S.R., Ranjbar M., Hasantabar V. (2020). Preventive effect of silymarin-loaded chitosan nanoparticles against global cerebral ischemia/reperfusion injury in rats. Eur. J. Pharmacol..

[121] Akbari-Kordkheyli V., Abbaszadeh-Goudarzi K., Nejati-Laskokalayeh M., Zarpou S., Khonakdar-Tarsi A. (2019). The protective effects of silymarin on ischemia-reperfusion injuries: A mechanistic review. Iran. J. Basic. Med. Sci..

[122] Zarpou S., Mosavi H., Bagheri A., Malekzadeh Shafaroudi M., Khonakdar-Tarsi A. (2021). NF-κB and NLRP3 gene expression changes during warm hepatic ischemia-reperfusion in rats with and without silibinin. Gastroenterol. Hepatol. Bed Bench..

[123] Pasala P.K., Uppara R.K., Rudrapal M., Zothantluanga J.H., Umar A.K. (2022). Silybin phytosome attenuates cerebral ischemia-reperfusion injury in rats by suppressing oxidative stress and reducing inflammatory response: In vivo and in silico approaches. J. Biochem. Mol. Toxicol..

[124] Kyriakopoulos G., Lambropoulou M., Valsami G., Kostomitsopoulos N., Konstandi O., Anagnostopoulos K., Tsalikidis C., Oikonomou P., Simopoulos K., Tsaroucha A.K. (2022). Pro-inflammatory cytokines/chemokines, TNF-α, IL-6 and MCP-1, as biomarkers for the nephro- and pneumoprotective effect of silibinin after hepatic ischemia/reperfusion: Confirmation by immunohistochemistry and qRT-PCR. Basic Clin. Pharmacol. Toxicol..

[125] Jin G., Bai D., Yin S., Yang Z., Zou D., Zhang Z., Li X., Sun Y., Zhu Q. (2016). Silibinin rescues learning and memory deficits by attenuating microglia activation and preventing neuroinflammatory reactions in SAMP8 mice. Neurosci. Lett..

[126] Kumar J., Park K.C., Awasthi A., Prasad B. (2015). Silymarin extends lifespan and reduces proteotoxicity in C. elegans Alzheimer’s model. CNS Neurol. Disord. Drug Targets.

[127] Sarubbo F., Ramis M.R., Kienzer C., Aparicio S., Esteban S., Miralles A., and Moranta D. (2018). Chronic silymarin, quercetin and naringenin treatments increase monoamines synthesis and hippocampal Sirt1 levels improving cognition in aged rats. J. Neuroimmune. Pharmacol..

[128] Vargas-Mendoza N., Ángeles-Valencia M., Madrigal-Santillán E.O., Morales-Martínez M., Tirado-Lule J.M., Solano-Urrusquieta A., Madrigal-Bujaidar E., Álvarez-González I., Fregoso-Aguilar T., Morales-González Á. (2020). Effect of silymarin supplementation on physical performance, muscle and myocardium histological changes, bodyweight, and food consumption in rats subjected to regular exercise training. Int. J. Mol. Sci..

[129] Vargas-Mendoza N., Angeles-Valencia M., Morales-González Á., Morales-Martínez M., Madrigal-Bujaidar E., Álvarez-González I., Fregoso-Aguilar T., Delgado-Olivares L., Madrigal-Santillán E.O., Morales-González J.A. (2021). Effect of silymarin supplementation in lung and liver histological modifications during exercise training in a rodent model. J. Funct. Morphol. Kinesiol..

[130] Aghaei F., Wong A., Zargani M., Sarshin A., Feizolahi F., Derakhshan Z., Hashemi M., Arabzadeh E. (2023). Effects of swimming exercise combined with silymarin and vitamin C supplementation on hepatic inflammation, oxidative stress, and histopathology in elderly rats with high-fat diet-induced liver damage. Nutrition.

[131] Kim S.H., Oh D.S., Oh J.Y., Son T.G., Yuk D.Y., Jung Y.S. (2016). Silymarin prevents restraint stress-induced acute liver injury by ameliorating oxidative stress and reducing inflammatory response. Molecules.

[132] Zhu Z., Sun G. (2018). Silymarin mitigates lung impairments in a rat model of acute respiratory distress syndrome. Inflammopharmacology.

[133] Garikapati D.R., Shaik P.B., Penchalaiah H. (2018). Evaluate neuroprotective effect of silibinin using chronic unpredictable stress (cus) model. Int. J. Physiol. Pathophysiol. Pharmacol..

[134] Son Y., Lee H.J., Rho J.K., Chung S.Y., Lee C.G., Yang K., Kim S.H., Lee M., Shin I.S., Kim J.S. (2015). The ameliorative effect of silibinin against radiation-induced lung injury: Protection of normal tissue without decreasing therapeutic efficacy in lung cancer. BMC Pulm. Med..

[135] Rigby C.M., Roy S., Deep G., Guillermo-Lagae R., Jain A.K., Dhar D., Orlicky D.J., Agarwal C., Agarwal R. (2017). Role of p53 in silibinin-mediated inhibition of ultraviolet B radiation-induced DNA damage, inflammation and skin carcino-genesis. Carcinogenesis.

[136] Ali Karami M., Sharif Makhmalzadeh B., Pooranian M., Rezai A. (2021). Preparation and optimization of silibinin-loaded chitosan-fucoidan hydrogel: An in vivo evaluation of skin protection against UVB. Pharm. Dev. Technol..

[137] Cha S.R., Jang J., Park S.M., Ryu S.M., Cho S.J., Yang S.R. (2023). Cigarette Smoke-Induced Respiratory Response: Insights into Cellular Processes and Biomarkers. Antioxidants.

[138] Ko J.W., Shin N.R., Park S.H., Lee I.C., Ryu J.M., Kim H.J., Cho Y.K., Kim J.C., Shin I.S. (2017). Silibinin inhibits the fibrotic responses induced by cigarette smoke via suppression of TGF-β1/Smad 2/3 signaling. Food Chem. Toxicol..

[139] Li D., Xu D., Wang T., Shen Y., Guo S., Zhang X., Guo L., Li X., Liu L., Wen F. (2015). Silymarin attenuates airway inflammation induced by cigarette smoke in mice. Inflammation.

[140] Park J.W., Shin N.R., Shin I.S., Kwon O.K., Kim J.S., Oh S.R., Kim J.H., Ahn K.S. (2016). Silibinin inhibits neutrophilic inflammation and mucus secretion induced by cigarette smoke via suppression of ERK-SP1 pathway. Phytother. Res..

[141] Ahmed R.F., Moussa R.A., Eldemerdash R.S., Zakaria M.M., Abdel-Gaber S.A. (2019). Ameliorative effects of silymarin on HCl-induced acute lung injury in rats; role of the Nrf-2/HO-1 pathway. Iran. J. Basic. Med. Sci..

[142] Hanafy N., El-Kemary M.A. (2022). Silymarin/curcumin loaded albumin nanoparticles coated by chitosan as muco-inhalable delivery system observing anti-inflammatory and anti COVID-19 characterizations in oleic acid triggered lung injury and in vitro COVID-19 experiment. Int. J. Biol. Macromol..

[143] Kosari-Nasab M., Shokouhi G., Ghorbanihaghjo A., Abbasi M.M., Salari A.A. (2018). Anxiolytic- and antidepressant-like effects of silymarin compared to diazepam and fluoxetine in a mouse model of mild traumatic brain injury. Toxicol. Appl. Pharmacol..

[144] Shokouhi G., Kosari-Nasab M., Salari A.A. (2020). Silymarin sex-dependently improves cognitive functions and alters TNF-α, BDNF, and glutamate in the hippocampus of mice with mild traumatic brain injury. Life Sci..

[145] Clichici S., David L., Moldovan B., Baldea I., Olteanu D., Filip M., Nagy A., Luca V., Crivii C., Mircea P. (2020). Hepatoprotective effects of silymarin coated gold nanoparticles in experimental cholestasis. Mater. Sci. Eng. C Mater. Biol. Appl..

[146] Nazemian F., Karimi G., Moatamedi M., Charkazi S., Shamsara J., Mohammadpour A.H. (2010). Effect of silymarin administration on TNF-α serum concentration in peritoneal dialysis patients. Phytother. Res..

[147] Atawia R.T., Mosli H.H., Tadros M.G., Khalifa A.E., Mosli H.A., Abdel-Naim A.B. (2014). Modulatory effect of silymarin on inflammatory mediators in experimentally induced benign prostatic hyperplasia: Emphasis on PTEN, HIF-1α, and NF-κB. Naunyn Schmiedebergs Arch. Pharmacol..

[148] Mazhari S., Razi M., Sadrkhanlou R. (2018). Silymarin and celecoxib ameliorate experimental varicocele-induced pathogenesis: Evidences for oxidative stress and inflammation inhibition. Int. Urol. Nephrol..

[149] Mady F.M., Essa H., El-Ammawi T., Abdelkader H., Hussein A.K. (2016). Formulation and clinical evaluation of silymarin pluronic-lecithin organogels for treatment of atopic dermatitis. Drug Des. Devel. Ther..

[150] Rigon C., Marchiori M., da Silva Jardim F., Pegoraro N.S., Chaves P., Velho M.C., Beck R., Ourique A.F., Sari M., Oliveira S.M. (2019). Hydrogel containing silibinin nanocapsules presents effective anti-inflammatory action in a model of irritant contact dermatitis in mice. Eur. J. Pharm. Sci..

[151] Choi Y.H., Jin G.Y., Guo H.S., Piao H.M., Li L.C., Li G.Z., Lin Z.H., Yan G.H. (2012). Silibinin attenuates allergic airway inflammation in mice. Biochem. Biophys. Res. Commun..

[152] Min K., Yoon W.K., Kim S.K., Kim B.H. (2007). Immunosuppressive effect of silibinin in experimental autoimmune encephalomyelitis. Arch. Pharm. Res..

[153] Toklu H.Z., Tunali Akbay T., Velioglu-Ogunc A., Ercan F., Gedik N., Keyer-Uysal M., Sener G. (2008). Silymarin, the antioxidant component of *Silybum marianum*, prevents sepsis-induced acute lung and brain injury. J. Surg. Res..

[154] Nasab E.M., Athari S.M., Ghafarzade S., Nasab A.M., Athari S.S. (2020). Immunomodulatory effects of two silymarin isomers in a Balb/c mouse model of allergic asthma. Allergol. Immunopathol..

[155] Navabi F., Shaygannejad V., Abbasirad F., Vaez E., Hosseininasab F., Kazemi M., Mirmosayyeb O., Alsahebfosoul F., Esmaeil N. (2019). Immunoregulatory effects of silymarin on proliferation and activation of Th1 cells isolated from newly diagnosed and IFN-ß_1b_-treated MS patients. Inflammation.

[156] Yang H.L., Shi X.W. (2021). Silybin alleviates experimental autoimmune encephalomyelitis by suppressing dendritic cell activation and Th17 cell differentiation. Front. Neurol..

[157] Ferraz A.C., Almeida L.T., da Silva Caetano C.C., da Silva Menegatto M.B., Souza Lima R.L., de Senna J., de Oliveira Cardoso J.M., Perucci L.O., Talvani A., Geraldo de Lima W. (2021). Hepatoprotective, antioxidant, anti-inflammatory, and antiviral activities of silymarin against mayaro virus infection. Antivir. Res..

[158] Stolf A.M., Cardoso C.C., Acco A. (2017). **2017**. Effects of silymarin on diabetes mellitus complications: A review. Phytother. Res..

[159] Chu C., Li D., Zhang S., Ikejima T., Jia Y., Wang D., Xu F. (2018). Role of silibinin in the management of diabetes mellitus and its complications. Arch. Pharm. Res..

[160] Lempesis I.G., Georgakopoulou V.E. (2023). Physiopathological mechanisms related to inflammation in obesity and type 2 diabetes mellitus. World J. Exp. Med..

[161] Stolf A.M., Campos Cardoso C., Morais H., Alves de Souza C.E., Lomba L.A., Brandt A.P., Agnes J.P., Collere F.C., Galindo C.M., Corso C.R. (2018). Effects of silymarin on angiogenesis and oxidative stress in streptozotocin-induced diabetes in mice. Biomed. Pharmacother..

[162] Chen Y., Chen L., Yang T. (2021). Silymarin nanoliposomes attenuate renal injury on diabetic nephropathy rats via co-suppressing TGF-β/Smad and JAK2/STAT3/SOCS1 pathway. Life Sci..

[163] Mohammadi H., Manouchehri H., Changizi R., Bootorabi F., Khorramizadeh M.R. (2020). Concurrent metformin and silibinin therapy in diabetes: Assessments in zebrafish (*Danio rerio*) animal model. J. Diabetes Metab. Disord..

[164] Zhang J., Shi Q., Hu Y., Li X. (2022). Silibinin augments the effect of clopidogrel on atherosclerosis in diabetic ApoE deficiency mice. Clin. Hemorheol. Microcirc..

[165] Wang C., Wang Z., Zhang X., Zhang X., Dong L., Xing Y., Li Y., Liu Z., Chen L., Qiao H. (2012). Protection by silibinin against experimental ischemic stroke: Up-regulated pAkt, pmTOR, HIF-1α and Bcl-2, down-regulated Bax, NF-κB expression. Neurosci. Lett..

[166] Li Z., Wang H., Yin Y. (2023). Peripheral inflammation is a potential etiological factor in Alzheimer’s disease. Rev. Neurosci..

[167] Wiatrak B., Jawień P., Szeląg A., Jęśkowiak-Kossakowska I. (2023). Does Inflammation Play a Major Role in the Pathogenesis of Alzheimer’s Disease?. Neuromolecular Med..

[168] Standaert D.G., Harms A.S., Childers G.M., Webster J.M. (2023). Disease mechanisms as subtypes: Inflammation in Parkinson disease and related disorders. Handb. Clin. Neurol..

[169] Williams G.P., Schonhoff A.M., Sette A., Lindestam Arlehamn C.S. (2022). Central and Peripheral Inflammation: Connecting the Immune Responses of Parkinson’s Disease. J. Park. Dis..

[170] Jung U.J., Jeon M.T., Choi M.S., Kim S.R. (2014). Silibinin attenuates MPP^+^-induced neurotoxicity in the substantia nigra in vivo. J. Med. Food.

[171] Huo Q., Shi Y., Qi Y., Huang L., Sui H., Zhao L. (2021). Biomimetic silibinin-loaded macrophage-derived exosomes induce dual inhibition of Aβ aggregation and astrocyte activation to alleviate cognitive impairment in a model of Alzheimer’s disease. Mater. Sci. Eng. C Mater. Biol. Appl..

[172] Leem E., Oh Y.S., Shin W.H., Jin B.K., Jeong J.Y., Shin M., Kim D.W., Jang J.H., Kim H.J., Ha C.M. (2019). Effects of silibinin against prothrombin Kringle-2-induced neurotoxicity in the nigrostriatal dopaminergic system In vivo. J. Med. Food.

[173] Ramírez-Carreto R.J., Zaldívar-Machorro V.J., Pérez-Ramírez D.J., Rodríguez-López B.E., Meza C., García E., Santamaría A., Chavarría A. (2023). Oral Administration of Silybin Protects Against MPTP-Induced Neurotoxicity by Reducing Pro-inflammatory Cytokines and Preserving BDNF Levels in Mice. Mol. Neurobiol..

[174] Karim A., Anwar F., Saleem U., Fatima S., Ismail T., Obaidullah A.J., Khayat R.O., Alqahtani M.J., Alsharif I., Khan H. (2023). Administration of α-lipoic acid and silymarin attenuates aggression by modulating endocrine, oxidative stress and inflammatory pathways in mice. Metab. Brain Dis..

[175] Habotta O., Ateya A., Saleh R.M., El-Ashry E.S. (2023). Thiamethoxam evoked neural oxido-inflammatory stress in male rats through modulation of Nrf2/NF-kB/iNOS signaling and inflammatory cytokines: Neuroprotective effect of Silymarin. Neurotoxicology.

[176] Haddadi R., Eyvari-Brooshghalan S., Makhdoomi S., Fadaiie A., Komaki A., Daneshvar A. (2023). Neuroprotective effects of silymarin in 3-nitropropionic acid-induced neurotoxicity in male mice: Improving behavioral deficits by attenuating oxidative stress and neuroinflammation. Naunyn Schmiedebergs Arch. Pharmacol..

[177] Herrock O., Deer E., LaMarca B. (2023). Setting a stage: Inflammation during preeclampsia and postpartum. Front. Physiol..

[178] Alston M.C., Redman L.M., Sones J.L. (2022). An Overview of Obesity, Cholesterol, and Systemic Inflammation in Preeclampsia. Nutrients.

[179] Giorgi V.S., Peracoli M.T., Peracoli J.C., Witkin S.S., Bannwart-Castro C.F. (2012). Silibinin modulates the NF-κB pathway and pro-inflammatory cytokine production by mononuclear cells from preeclamptic women. J. Reprod. Immunol..

[180] Cristofalo R., Bannwart-Castro C.F., Magalhães C.G., Borges V.T., Peraçoli J.C., Witkin S.S., Peraçoli M.T. (2013). Silibinin attenuates oxidative metabolism and cytokine production by monocytes from preeclamptic women. Free Rad. Res..

[181] Lim R., Morwood C.J., Barker G., Lappas M. (2014). Effect of silibinin in reducing inflammatory pathways in in vitro and in vivo models of infection-induced preterm birth. PLoS ONE.

[182] Ribeiro V.R., Romao-Veiga M., Nunes P.R., de Oliveira L., Romagnoli G.G., Peracoli J.C., Peracoli M. (2022). Silibinin downregulates the expression of the Th1 and Th17 profiles by modulation of STATs and transcription factors in pregnant women with preeclampsia. Int. Immunopharmacol..

[183] Gluba-Brzózka A., Franczyk B., Rysz-Górzyńska M., Rokicki R., Koziarska-Rościszewska M., Rysz J. (2021). Pathomechanisms of Immunological Disturbances in β-Thalassemia. Int. J. Mol. Sci..

[184] Balouchi S., Gharagozloo M., Esmaeil N., Mirmoghtadaei M., Moayedi B. (2014). Serum levels of TGFβ, IL-10, IL-17, and IL-23 cytokines in β-thalassemia major patients: The impact of silymarin therapy. Immunopharmacol. Immunotoxicol..

[185] Alidoost F., Gharagozloo M., Bagherpour B., Jafarian A., Sajjadi S.E., Hourfar H., Moayedi B. (2006). Effects of silymarin on the proliferation and glutathione levels of peripheral blood mononuclear cells from beta-thalassemia major patients. International Immunopharmacology.

[186] Darvishi-Khezri H., Kosaryan M., Karami H., Salehifar E., Mahdavi M., Alipour A., Aliasgharian A. (2021). Can use of silymarin improve inflammatory status in patients with β-Thalassemia Major? A crossover, randomized controlled trial. Complement. Med. Res..

[187] Uçmak F., Ekin N., İbiloğlu İ., Arslan S., Kaplan İ., Şenateş E. (2016). Prophylactic administration of silybin ameliorates L-Arginine-induced acute pancreatitis. Med. Sci. Monit..

[188] Song J., Pan W., Sun Y., Han J., Shi W., Liao W. (2017). Aspergillus fumigatus-induced early inflammatory response in pulmonary microvascular endothelial cells: Role of p38 MAPK and inhibition by silibinin. Int. Immunopharmacol..

[189] Ali S.A., Saifi M.A., Godugu C., Talla V. (2021). Silibinin alleviates silica-induced pulmonary fibrosis: Potential role in modulating inflammation and epithelial-mesenchymal transition. Phytother. Res..

[190] Hussain S.A., Jassim N.A., Numan I.T., Al-Khalifa I.I., Abdullah T.A. (2009). Anti-inflammatory activity of silymarin in patients with knee osteoarthritis. A comparative study with piroxicam and meloxicam. Saudi Med. J..

[191] Arafa Keshk W., Zahran S.M., Katary M.A., Abd-Elaziz Ali D. (2017). Modulatory effect of silymarin on nuclear factor-erythroid-2-related factor 2 regulated redox status, nuclear factor-κB mediated inflammation and apoptosis in experimental gastric ulcer. Chem. Biol. Interact..

[192] Esmaily H., Vaziri-Bami A., Miroliaee A.E., Baeeri M., Abdollahi M. (2011). The correlation between NF-κB inhibition and disease activity by coadministration of silibinin and ursodeoxycholic acid in experimental colitis. Fundam. Clin. Pharmacol..

[193] Safarpour S., Safarpour S., Moghadamnia A.A., Kazemi S., Ebrahimpour A., Shirafkan F. (2023). The protective effects of silymarin nanoemulsion on 5-fluorouracil-induced gastrointestinal toxicity in rats. Saudi Pharm. J..

[194] Akhtar M., Chen Y., Ma Z., Zhang X., Shi D., Khan J.A., Liu H. (2021). Gut microbiota-derived short chain fatty acids are potential mediators in gut inflammation. Anim. Nutr..

[195] Oryan A., Tabatabaei Naeini A., Moshiri A., Mohammadalipour A., Tabandeh M.R. (2012). Modulation of cutaneous wound healing by silymarin in rats. J. Wound Care.

[196] Sharifi R., Rastegar H., Kamalinejad M., Dehpour A.R., Tavangar S.M., Paknejad M., Mehrabani Natanzi M., Ghannadian N., Akbari M., Pasalar P. (2012). Effect of topical application of silymarin (*Silybum marianum*) on excision wound healing in albino rats. Acta Med. Iran..

[197] Mahmoodi-Nesheli M., Alizadeh S., Solhi H., Mohseni J., Mahmoodi-Nesheli M. (2018). Adjuvant effect of oral silymarin on patients’ wound healing process caused by thermal injuries. Casp. J. Intern. Med..

[198] Liu X., Xu Q., Long X., Liu W., Zhao Y., Hayashi T., Hattori S., Fujisaki H., Ogura T., Tashiro S.I. (2019). Silibinin-induced autophagy mediated by PPARα-sirt1-AMPK pathway participated in the regulation of type I collagen-enhanced migration in murine 3T3-L1 preadipocytes. Mol. Cell. Biochem..

[199] Soleimani H., Behfar M., Hobbenaghi R. (2021). Tenogenic effects of silymarin following experimental Achilles tendon transection in rats. Vet. Res. Forum.

[200] Gupta S.C., Tyagi A.K., Deshmukh-Taskar P., Hinojosa M., Prasad S., Aggarwal B.B. (2014). Downregulation of tumor necrosis factor and other proinflammatory biomarkers by polyphenols. Arch. Biochem. Biophys..

[201] Stevenson D.E., Hurst R.D. (2007). Polyphenolic phytochemicals–just antioxidants or much more?. Cell. Mol. Life Sci..

[202] Manna S.K., Mukhopadhyay A., Van N.T., Aggarwal B.B. (1999). Silymarin suppresses TNF-induced activation of NF-κB, c-Jun N-terminal kinase, and apoptosis. J. Immunol..

[203] Gharagozloo M., Velardi E., Bruscoli S., Agostini M., Di Sante M., Donato V., Amirghofran Z., Riccardi C. (2010). Silymarin suppress CD4+ T cell activation and proliferation: Effects on NF-kappaB activity and IL-2 production. Pharm. Res..

[204] Oskoueian E., Abdullah N., Idrus Z., Ebrahimi M., Goh Y.M., Shakeri M., Oskoueian A. (2014). Palm kernel cake extract exerts hepatoprotective activity in heat-induced oxidative stress in chicken hepatocytes. BMC Complement. Altern. Med..

[205] Kang D.Y., Sp N., Do Park K., Lee H.K., Song K.D., Yang Y.M. (2019). Silibinin inhibits in vitro ketosis by regulating HMGCS2 and NF-κB: Elucidation of signaling molecule relationship under ketotic conditions. In vitro cellular & developmental biology. Animal.

[206] Xie Y., Feng S.L., Mai C.T., Zheng Y.F., Wang H., Liu Z.Q., Zhou H., Liu L. (2021). Suppression of up-regulated LXRα by silybin ameliorates experimental rheumatoid arthritis and abnormal lipid metabolism. Phytomedicine.

[207] Baldini F., Portincasa P., Grasselli E., Damonte G., Salis A., Bonomo M., Florio M., Serale N., Voci A., Gena P. (2020). Aquaporin-9 is involved in the lipid-lowering activity of the nutraceutical silybin on hepatocytes through modulation of autophagy and lipid droplets composition. Biochim. Biophys. Acta Mol. Cell. Biol. Lipids.

[208] Tian M.Y., Fan J.H., Zhuang Z.W., Dai F., Wang C.Y., Hou H.T., Ma Y.Z. (2019). Effects of silymarin on p65 NF-κB, p38 MAPK and CYP450 in LPS-induced hoof dermal inflammatory cells of dairy cows. BMC Vet. Res..

[209] Prack Mc Cormick B., Langle Y., Belgorosky D., Vanzulli S., Balarino N., Sandes E., Eiján A.M. (2018). Flavonoid silybin improves the response to radiotherapy in invasive bladder cancer. J. Cell. Biochem..

[210] Liu X., Xu Q., Liu W., Yao G., Zhao Y., Xu F., Hayashi T., Fujisaki H., Hattori S., Tashiro S.I. (2018). Enhanced migration of murine fibroblast-like 3T3-L1 preadipocytes on type I collagen-coated dish is reversed by silibinin treatment. Mol. Cell. Biochem..

[211] Henriet E., Abdallah F., Laurent Y., Guimpied C., Clement E., Simon M., Pichon C., Baril P. (2022). Targeting TGF-β1/miR-21 Pathway in Keratinocytes Reveals Protective Effects of Silymarin on Imiquimod-Induced Psoriasis Mouse Model. JID Innov..

[212] Wang Q., Zou L., Liu W., Hao W., Tashiro S.I., Onodera S., Ikejima T. (2011). Inhibiting NF-κB activation and ROS production are involved in the mechanism of silibinin’s protection against D-galactose-induced senescence. Pharmacol. Biochem. Behav..

[213] Prabu S.M., Muthumani M. (2012). Silibinin ameliorates arsenic induced nephrotoxicity by abrogation of oxidative stress, inflammation and apoptosis in rats. Mol. Biol. Rep..

[214] Salamone F., Galvano F., Cappello F., Mangiameli A., Barbagallo I., Li Volti G. (2012). Silibinin modulates lipid homeostasis and inhibits nuclear factor kappa B activation in experimental nonalcoholic steatohepatitis. Transl. Res..

[215] Li C.C., Hsiang C.Y., Wu S.L., Ho T.Y. (2012). Identification of novel mechanisms of silymarin on the carbon tetrachloride-induced liver fibrosis in mice by nuclear factor-κB bioluminescent imaging-guided transcriptomic analysis. Food Chem. Toxicol..

[216] Liu B.N., Han B.X., Liu F. (2014). Neuroprotective effect of pAkt and HIF-1α on ischemia rats. Asian Pac. J. Trop. Med..

[217] Aristatile B., Al-Assaf A.H., Pugalendi K.V. (2013). Carvacrol suppresses the expression of inflammatory marker genes in D-galactosamine-hepatotoxic rats. Asian Pac. J. Trop. Med..

[218] Salama S.M., Abdulla M.A., Al Rashdi A.S., Hadi A.H.A. (2013). Mechanism of hepatoprotective effect of Boesenbergia rotunda in thioacetamide-induced liver damage in rats. Evid. Based Complement. Alternat. Med..

[219] Abhilash P.A., Harikrishnan R., Indira M. (2013). Ascorbic acid is superior to silymarin in the recovery of ethanol-induced inflammatory reactions in hepatocytes of guinea pigs. J. Physiol. Biochem..

[220] Abhilash P.A., Harikrishnan R., Indira M. (2014). Ascorbic acid suppresses endotoxemia and NF-κB signaling cascade in alcoholic liver fibrosis in guinea pigs: A mechanistic approach. Toxicol. Appl. Pharmacol..

[221] Sharma S., Le Guillou D., Chen J.Y. (2023). Cellular stress in the pathogenesis of nonalcoholic steatohepatitis and liver fibrosis. Nat. Rev. Gastroenterol. Hepatol..

[222] Liu K., Zhou S., Liu J., Wang Y., Zhu F., Liu M. (2019). Silibinin attenuates high-fat diet-induced renal fibrosis of diabetic nephropathy. Drug Des. Devel. Ther..

[223] Ali N.M., Mahmoud A.A.A., Mahmoud M.F., El Fayoumi H.M. (2019). Glycyrrhizic acid and silymarin alleviate the neurotoxic effects of aluminum in rats challenged with fructose-induced insulin resistance: Possible role of toll-like receptor 4 pathway. Drug Chem. Toxicol..

[224] El-Marasy S.A., Abd-Elsalam R.M., Ahmed-Farid O.A. (2018). Ameliorative Effect of Silymarin on Scopolamine-induced Dementia in Rats. Open Access Maced. J. Med. Sci..

[225] Yuan R., Fan H., Cheng S., Gao W., Xu X., Lv S., Ye M., Wu M., Zhu X., Zhang Y. (2017). Silymarin prevents NLRP3 inflammasome activation and protects against intracerebral hemorrhage. Biomed. Pharmacother..

[226] Tian L., Li W., Wang T. (2017). Therapeutic effects of silibinin on LPS-induced acute lung injury by inhibiting NLRP3 and NF-κB signaling pathways. Microb. Pathog..

[227] Marcolino Assis-Júnior E., Melo A.T., Pereira V.B.M., Wong D.V.T., Sousa N.R.P., Oliveira C.M.G., Malveira L.R.C., Moreira L.S., Souza M.H.L.P., Almeida P.R.C. (2017). Dual effect of silymarin on experimental non-alcoholic steatohepatitis induced by irinotecan. Toxicol. Appl. Pharmacol..

[228] Liu P., Chen W., Kang Y., Wang C., Wang X., Liu W., Hayashi T., Qiu Z., Mizuno K., Hattori S. (2023). Silibinin ameliorates STING-mediated neuroinflammation via downregulation of ferroptotic damage in a sporadic Alzheimer’s disease model. Arch. Biochem. Biophys..

[229] Karimi R., Bakhshi A., Dayati P., Abazari O., Shahidi M., Savaee M., Kafi E., Rahmanian M., Naghib S.M. (2022). Silymarin reduces retinal microvascular damage in streptozotocin-induced diabetic rats. Sci. Rep..

[230] Menzel A., Samouda H., Dohet F., Loap S., Ellulu M.S., Bohn T. (2021). Common and novel markers for measuring inflammation and oxidative stress Ex Vivo in research and clinical practice-Which to use regarding disease outcomes?. Antioxidants.

[231] Cardoso Dal Pont G., Farnell M., Farnell Y., Kogut M.H. (2020). Dietary factors as triggers of low-grade chronic intestinal inflammation in poultry. Microorganisms.

[232] Kogut M.H., Genovese K., Swaggerty C.L., He H., Broom L. (2018). Inflammatory phenotypes in the intestine of poultry: Not all inflammation is created equal. Poult. Sci..

[233] Ducatelle R., Goossens E., Eeckhaut V., Van Immerseel F. (2023). Poultry gut health and beyond. Anim. Nutr..

[234] Tellez-Isaias G., Eisenreich W., Petrone-Garcia V.M., Hernandez-Velasco X., Castellanos-Huerta I., Tellez G., Latorre J.D., Bottje W.G., Senas-Cuesta R., Coles M.E. (2023). Effects of chronic stress and intestinal inflammation on commercial poultry health and performance: A review. Ger. J. Vet. Res..

[235] Gusev E., Zhuravleva Y. (2022). Inflammation: A new look at an old problem. Int. J. Mol. Sci..

[236] Ralli T., Tripathi T., Kalaiselvan V., Tiwari R., Aeri V., Kohli K. (2023). Silymarin as a Phyto-pharmaceutical: Isolation, Simultaneous Quantification of four Biomarkers and in-silico Anti-inflammatory Activity. Chin. J. Anal. Chem..

[237] Yu C., Wang D., Yang Z., Wang T. (2022). Pharmacological effects of polyphenol phytochemicals on the intestinal inflammation via targeting TLR4/NF-κB signaling pathway. Int. J. Mol. Sci..

[238] DuBreuil D.M., Lai X., Zhu K., Chahyadinata G., Perner C., Chiang B.M., Battenberg A., Sokol C.L., Wainger B.J. (2023). Phenotypic screen identifies the natural product silymarin as a novel anti-inflammatory analgesic. Mol. Pain.

[239] Ghodousi M., Karbasforooshan H., Arabi L., Elyasi S. (2023). Silymarin as a preventive or therapeutic measure for chemotherapy and radiotherapy-induced adverse reactions: A comprehensive review of preclinical and clinical data. Eur. J. Clin. Pharm..

[240] Gür F.M., Bilgiç S. (2023). Silymarin, an antioxidant flavonoid, protects the liver from the toxicity of the anticancer drug paclitaxel. Tissue Cell.

